# Clinical and cost effectiveness of a corticosteroid injection versus exercise therapy for shoulder pain in general practice: protocol for a randomised controlled trial (SIX Study)

**DOI:** 10.1136/bmjopen-2021-050101

**Published:** 2021-03-30

**Authors:** Pieter F van Doorn, Evelien I T de Schepper, Dieuwke Schiphof, Ramon P G Ottenheijm, Marloes Thoomes-de Graaf, Marc A Koopmanschap, John M van Ochten, Danielle A van der Windt, Patrick J E Bindels, Bart W Koes, Jos Runhaar

**Affiliations:** 1 General Practice, Erasmus MC, Rotterdam, The Netherlands; 2 Family Medicine, Maastricht University, Maastricht, The Netherlands; 3 Fysio-Experts, Hazerswoude Rijndijk, The Netherlands; 4 Health Economics and HTA, Erasmus University Rotterdam, Rotterdam, The Netherlands; 5 Primary Care Musculoskeletal Research Centre, Keele University, Keele, UK

**Keywords:** primary care, shoulder, clinical trials

## Abstract

**Introduction:**

Shoulder pain is common and the prognosis is often unfavourable. Dutch guidelines on the treatment of shoulder pain in primary care recommend a corticosteroid injection or a referral to exercise therapy, if initial pain management fails and pain persists. However, evidence of the effectiveness of a corticosteroid injection compared with exercise therapy, especially in the long term, is limited. This trial will assess the clinical effectiveness and cost effectiveness of a corticosteroid injection compared with physiotherapist-led exercise therapy over 12 months follow-up in patients with shoulder pain in primary care.

**Methods and analysis:**

The SIX Study is a multicentre, pragmatic randomised clinical trial in primary care. A total of 213 patients with shoulder pain, aged ≥18 years presenting in general practice will be included. Patients will be randomised (1:1) into two groups: a corticosteroid injection or 12 sessions of physiotherapist-led exercise therapy. The effect of the allocated treatment will be assessed through questionnaires at 6 weeks and after 3, 6, 9 and 12 months. The primary outcome is patient’s reported shoulder pain-intensity and function, measured with the Shoulder Pain and Disability Index, over 12 months follow-up. Secondary outcomes include cost effectiveness, pain-intensity, function, health-related quality of life, sleep quality, patient’s global perceived effect, work absence, healthcare utilisation and adverse events. Between group differences will be evaluated using a repeated measurements analysis with linear effects models. A cost-utility analysis will be performed to assess the cost effectiveness using quality-adjusted life years from a medical and societal perspective.

**Ethics and dissemination:**

This study was approved by the Medical Ethics Committee of Erasmus MC University Medical Center Rotterdam (MEC 2020-0300). All participants will give written informed consent prior to data collection. The results from this study will be disseminated in international journals and implemented in the primary care guidelines on shoulder pain.

**Trial registration number:**

Dutch Trial Registry (NL8854).

**Strengths and limitations of this study**

- This is a large pragmatic randomised controlled trial that aims to evaluate two treatment options recommended by the guidelines in the management of persistent shoulder pain in general practice, a corticosteroid injection compared with exercise therapy.
- In addition to the clinical effectiveness, a cost-effectiveness analysis will be performed for both treatments.
- This study has a long follow-up period of 12 months, allowing for the analysis of the long-term (cost-)effectiveness of both treatments.
- The pragmatic nature of this trial has its drawbacks, however, it will provide a true reflection of both treatments applied in current practice.

## Introduction

### Background and rationale

Shoulder pain is the third most common musculoskeletal complaint in primary healthcare.^1–3^ The estimated incidence is reported at 30.3 per 1000 person-years.^3^ The prognosis for shoulder pain is often unfavourable. Only 50% of people presenting with a new episode of shoulder pain in primary care show complete recovery within 6 months.^4^ In general, apart from pain, patients with shoulder pain report having functional limitations which can reach a level of severity whereby they preclude work-related tasks.^5^ Work absence and treatment of shoulder pain generate high costs to society and healthcare.^6 7^ A recent cost-estimation study for patients with shoulder pain consulting in primary care in Sweden estimated the mean annual costs at €4139 per patient, with sick leave accounting for more than 80% of the total costs.^7^

Guidelines for the management of shoulder pain provide treatment options based on the initial diagnosis of the general practitioner (GP) and the severity of the pain.^8 9^ The recommended management options in the guidelines are focused on controlling pain and restoring or maintaining shoulder function. The recently updated primary care guideline for shoulder pain, issued by the Dutch College of General Practitioners (NHG) in 2019, recommends a stepped-care approach. In the first step, GPs are advised to start the treatment with advice and, if necessary, prescribe analgesics. If pain persists, the GP is recommended to either prolong or adjust analgesics, administer a local corticosteroid injection in case of severe pain or refer the patient to a physiotherapist for exercise therapy in case of (impending) dysfunction.^8^ Although the guideline recommends exercise therapy or corticosteroid injection when shoulder pain persists, the guideline acknowledges the lack of evidence to favour one option over the other.

A systematic review by Steuri *et al* on randomised controlled trials (RCTs) comparing corticosteroid injection(s) to exercise therapy showed that injections have statistically significant, but small effect on pain in the short term, generally within 6 weeks after the intervention, but not at longer follow-up time intervals (3–6 months).^10^ Given the low quality of most of the included studies and high level of heterogeneity, the authors concluded that larger, high quality trials are required. Moreover, the authors call for health economic evaluations alongside such trials to assess comparative cost effectiveness and cost utility. A similar call came from the Cochrane review by Page *et al* on manual therapy and exercise for rotator cuff disease; ‘high quality RCTs are needed to establish the benefits and harms of exercise interventions that reflect actual practice, compared with placebo, no intervention or active interventions with evidence of benefit (eg, glucocorticoid injection)’.^11^

Given the high incidence and costs associated with shoulder pain and the lack of high quality evidence to underpin current clinical practice and guideline recommendations, the recently published National Research Agenda by the NHG listed research on the effectiveness of corticosteroid injections for shoulder pain in general practice as a top priority.^12^ We have therefore designed an RCT to compare the clinical and cost effectiveness of corticosteroid injections and physiotherapist-led exercise therapy as primary care management interventions for patients with shoulder pain.

### Objectives

The primary objective of the Shoulder Injection and eXercise (SIX) trial is to compare the clinical effectiveness of a local corticosteroid injection to physiotherapist-led exercise therapy for shoulder pain in primary care over 12 months of follow-up. The main secondary objective is to compare the cost effectiveness of both treatments over a 12-month follow-up period.

## Methods and analysis

### Trial design/study setting

The study is a randomised, multicentre, open label, parallel group, pragmatic clinical trial. Patients will be recruited in Dutch general practices. GPs will select patients presented with shoulder pain who are suitable for both a local corticosteroid injection and physiotherapist-led exercise therapy. GPs will refer these patients to the SIX research team, who will further assess all potential patients for eligibility and will undertake informed consent procedures.

### Eligibility criteria

#### Inclusion criteria

- Patient has contacted their GP with shoulder pain due to subacromial pain syndrome or glenohumeral disorders.
- Aged 18 years or older.
- Qualified for both a local corticosteroid injection and physiotherapist-led exercise therapy, as indicated by the GP.
- Able to understand spoken and written Dutch language.

#### Exclusion criteria

- Shoulder pain due to recent serious trauma, malignancy, systemic rheumatologically disease, neurological or cardiac disease.^8^
- Shoulder pain due to instability of the glenohumeral joint, disorders of the acromioclavicular or sternoclavicular joint, or neck pain with additional shoulder pain.
- Treatment of the affected shoulder with corticosteroid injection or physiotherapy in the last 6 months.
- A history of serious shoulder trauma, such as fractures, ruptures, luxation or surgery.
- Contraindications for corticosteroid injection.
- Current use of oral corticosteroids.

For participants with bilateral shoulder pain, the most painful shoulder will be taken as the study shoulder.

### Parallel cohort study

Patients with shoulder pain who are not eligible for trial participation or patients who are eligible but do not want to be randomised, for example, due to strong treatment preferences, will be invited to participate in a parallel cohort study. With their consent, these patients will be assessed using the same outcome measures at similar time points. In addition, these patients will complete a questionnaire regarding their treatment preferences and reasons for not wanting to participate in the trial (if applicable) at baseline. This information will provide important information regarding the recruitment process by indicating if and why recruitment may be suboptimal or failing. Furthermore, the parallel cohort study will provide the unique possibility to compare baseline characteristics of randomised participants to those who were not eligible or not willing to be randomised and outcomes following their (preferred) treatment.

We anticipate recruiting around 600 patients to this parallel cohort study. All cohort participants who are not eligible for the RCT will be informed that if the initial GP treatment fails and they consider re-consultation, they are potentially eligible for the RCT. They can contact the SIX research team for receiving additional information regarding the trial and to initiate the consent procedure for the trial.

### Interventions

#### Corticosteroid injection

The corticosteroid injection will be delivered by the GP. The corticosteroid injection will consist of 40 mg triamcinolone acetonide (Kenacort-A 40), possibly in combination with a local anaesthetic agent, lidocaine 10 mg, at the discretion of the GP in accordance with the NHG guideline for shoulder pain.^8^

The site of the injection, subacromial or intra-articular, will depend on the initial diagnosis of the GP. Subacromial injections will be administered to participants diagnosed with subacromial pain syndrome and intra-articular injections on participants with glenohumeral joint pain. GPs are advised to follow the instructional videos on subacromial and intra-articular injection published by the NHG.^13^ All participating GPs will be invited for an optional shoulder injection training by an experienced doctor of orthopaedic medicine at the Erasmus MC.

Consultations with the GP will be coordinated so that participants typically receive their injection within 1 week of randomisation. In line with the guideline, a maximum of two injections will be permitted per patient, with the second injection, when considered necessary, offered 2–4 weeks after the first injection. Any participant receiving a second injection will have the date of administration recorded in their case report form.

#### Physiotherapist-led exercise therapy

Participants randomised to physiotherapist-led exercise therapy will be referred to one of the local physiotherapists. Preferably the physiotherapist is affiliated with the Dutch Shoulder Network (SNN). The SNN is an umbrella organisation for regional shoulder networks of physiotherapist practices. All affiliated physiotherapists have to complete a 2-day entry course on shoulder pain, accredited by the Royal Dutch Society for Physiotherapy (KNGF).

The exercise therapy will consist of a maximum of 12 treatments of around 30 min under the supervision of the physiotherapist over a course of 12–14 weeks. In addition, all participants will receive home-based exercise at the discretion of the physiotherapist. The intensity of the exercise is based on tissue irritability and the capacity of the patient. Pain during or after exercise is allowed, as long as there is no night-time pain and the pain returns to pre-training levels within 24 hours. Physiotherapist will be requested not to use massage, laser therapy, ultrasound therapy, transcutaneous electrical nerve stimulation, dry needling or acupuncture, given lack of evidence for effectiveness.^14^ All participating physiotherapists will receive a brief guideline developed in cooperation with the SNN describing the criteria for exercise therapy.

#### Co-interventions

This is a pragmatic clinical trial designed to evaluate the effectiveness of corticosteroid injections compared with physiotherapist-led exercise therapy for shoulder pain in real-life routine practice conditions. Therefore, participants will be instructed to continue their usual medication as discussed with their GP. Co-interventions after randomisation will be allowed and will be monitored through medical record review and questionnaires. This includes cross-over between interventions, which is estimated to occur in 20% of participants based on the number of patients receiving an injection and referral for exercise therapy in the ‘usual care’ treatment arm of a recent RCT.^15^

### Outcomes

Table 1 shows an overview of the time schedule of enrolment, interventions and all assessments for participants. The selection of outcome measures has been based on the core outcome set published by The Outcome Measures in Rheumatology (OMERACT) Shoulder Working Group.^16^ The primary outcome is shoulder pain-intensity and function measured using the Shoulder Pain and Disability Index (SPADI) total score over 12 months post randomisation.^17^ The SPADI is the most commonly used measure to assess pain-intensity and disability.^18^ The Dutch version of the SPADI has good psychometric properties.^19 20^

**Table 1:** Time schedule for enrolment, interventions and assessments for participants

Time point	Pre- randomisation	Baseline (T0)	6 weeks (T1)	3 months (T2)	6–9–12 months (T3–T4–T5)*
Enrolment					
Diagnosis	X				
Eligibility screening	X				
Informed consent	X				
Randomisation†		X			
Interventions					
Corticosteroid injection†		X			
Physiotherapist-led exercise therapy†		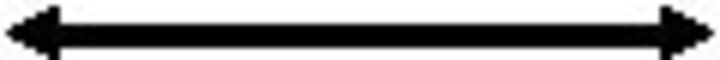			
Assessments					
Sociodemographics		X			
Current shoulder episode (location, duration, cause, course, stiffness)		X			
Previous shoulder episodes (history, treatments)		X			
Other current pain locations (pain manikin)		X			
Other relevant medical issues		X			
Psychological prognostic factors (HADS, FABQ)		X			
Current medical use for the shoulder pain		X			
Treatment preferences		X			
Treatment expectations		X			
Outcomes					
Pain and function (SPADI)		X	X	X	X
Medical costs (MCQ)			X	X	X
Global perceived effect (GPE)			X	X	X
Productivity costs (PCQ)			X	X	X
Health-related quality of life (EQ-5D-5L)		X	X	X	X
Sleep quality (SQS)		X	X	X	X
Side effects			X	X	
Serious adverse events (SAEs)			X	X	X

*At these time points the indicated outcome measures will be repeated.

†Randomisation occurs after baseline measurements are taken.

EQ-5D-5L, five-level version of the well-validated EuroQol Five-Dimensional Questionnaire; FABQ, Fear-avoidance Beliefs Questionnaire; HADS, Hospital Anxiety and Depression Scale; MCQ, Medical Cost Questionnaire; PCQ, Productivity Cost Questionnaire; SPADI, Shoulder Pain and Disability Index; SQS, Sleep Quality Scale.

Secondary outcomes include incremental costs per quality-adjusted life year (QALY) gained, using both the medical as well as the societal perspective, over 12 months post randomisation. Medical costs will be measured using the Medical Cost Questionnaire (MCQ) and societal costs will be measured using the Productivity Cost Questionnaire (PCQ).^21^ QALY will be measured using the five-level version of the well-validated EuroQol Five-Dimensional Questionnaire (EQ-5D-5L) score.^22^

Other secondary outcomes will be clinical effectiveness and cost effectiveness of the randomised treatments in the short term (6 weeks, 3 months) and medium term (6 months, 9 months). In addition, secondary outcomes will include subdomains (pain and function) of the SPADI, health-related quality of life (EQ-5D-5L), sleep quality measured with the Sleep Quality Scale,^23^ participant’s perceived recovery using the global perceived effect questionnaire,^16^ work absence as measured by the PCQ, healthcare utilisation as measured by the MCQ, side effects assessed at short term post randomisation and serious adverse events (SAEs) occurring post randomisation.

### Sample size

The target sample size is 85 participants in each trial group. This is based on 90% power and a 0.05 two-sided statistical significance to detect a minimally clinically important difference of ten points on the SPADI total scale,^24^ using a conservative estimation of a baseline SD of 20.^25^ Accounting for a potential loss to follow-up at 12 months of 20%, this will require a total of 213 patients to be randomised to the intervention groups.

### Recruitment

All patients (≥18 years old) consulting their GP for shoulder pain who are suitable for both a local corticosteroid injection and physiotherapist-led exercise therapy can be invited by their GP to participate in this study. These patients will be informed on the trial by the GP and are advised to contact the research team. The research team will provide further information on the trial and if the patient confirms their interest to participate in the trial, eligibility will be checked and the informed consent procedure will be completed. After the participant has completed the baseline questionnaire, the patient will be randomised by the research team. The patient and the GP will be notified on the randomisation result by the research ream.

**Figure 1:**
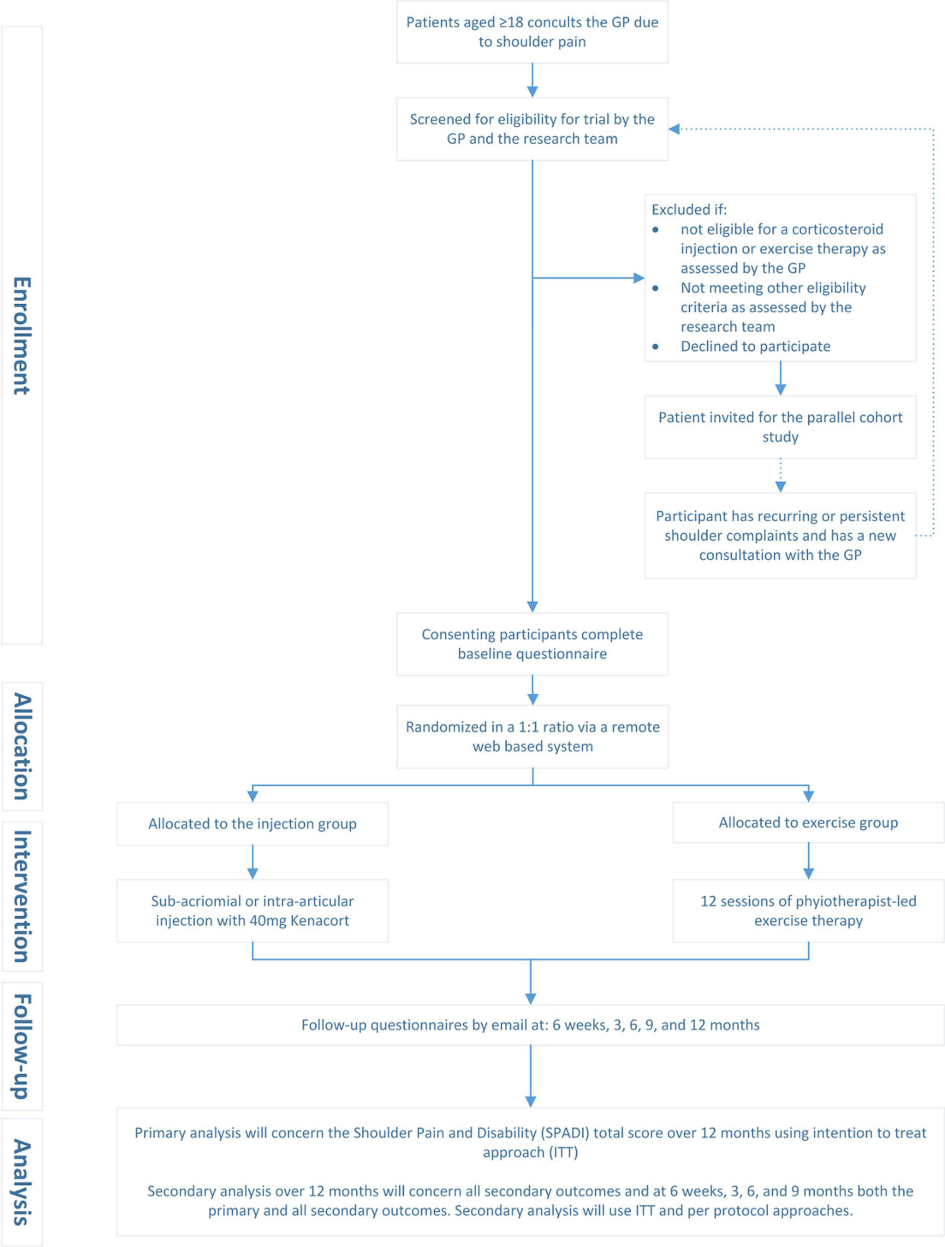
Consort flowchart of recruitment. GP, general practitioner.

All other patients (eg, wait-and-see policy or prescription of analgesics) will be invited to participate in the parallel cohort study. These patients will be invited through two weekly searches of the medical records of participating GP practices. All cohort participants will be informed that if the initial GP treatment fails and they consider re-consultation, they are potentially eligible for the RCT (figure 1).

Accounting for a 25% willingness of patients to participate in the RCT and in the cohort, a 50% willingness of participants in the cohort to enter the trial if initial treatment fails and a 25% loss due to not eligible for the trial, 2430 patients need to be invited to participate in either the trial or the cohort over the 18 months period. On average, a fulltime Dutch GP has around 2095 registered patients.^26^ With an incidence rate of 30.3 per 1000 person-years in the Netherlands,^3^ it is expected that a fulltime GP sees around six patients with a new episode of shoulder pain per month, which results in 23 GPs needed for this study. However, taking in account Lasagna’s law,^27^ we expect to need at least 46 GPs to ensure the total sample size.

### Allocation

The Erasmus MC Clinical Trial Center, who will not meet or contact the patients will prepare a remote web-based randomisation system using random blocks of 8, 6 or 4 to ensure concealment of allocation. Participants and their GP will be informed about the outcome of the randomisation by phone and participants will receive a patient card through mail detailing their treatment allocation and related procedures.

### Blinding

The researcher who will carry out the primary analysis will be blinded for treatment allocation. The participant and the GP will not be blinded for treatment allocation. This is not feasible in this pragmatic trial.

### Data collection methods

After obtaining informed consent, participants will complete the baseline questionnaire. Subsequently, the participants will be randomised to one of the two interventions groups. Participants will be asked to complete online questionnaires sent by email, after 6 weeks, 3, 6, 9 and 12 months after randomisation. If the follow-up questionnaire is not returned within 2 weeks of initial mailing, a reminder will be sent encouraging the participant to complete the questionnaire. Non-responders or responders with incomplete questionnaires will be contacted by telephone to pose them the missing questions.

### Data management

Data management will be performed via a web-based medical survey tracker (Gemstracker). Each participant will be allocated a unique code, which will be used on all trial-specific documents, except for the signed informed consent and contact details. Participants’ identifiable data will be stored separately and securely from study data in accordance with local procedures.

### Statistical methods

Baseline characteristics will be summarised using descriptive statistics. All analyses will be performed under intention-to-treat.

#### Primary analyses

The primary clinical outcome is patient reported severity of pain and function over 12 months post randomisation, measured with the SPADI total score. A linear mixed model with repeated measures will be used to generate estimates of effects. The time points included in this model will be baseline, 6 weeks, 3, 6, 9 and 12 months. Baseline values for the primary outcome are retained as part of the outcome vector and group means on the primary outcome are assumed to be equal at baseline (ie, an intervention-effect is restricted at baseline). Fixed effects will be time and time by treatment group. To model the covariance of repeated measures by participant, the option for data structure in the analyses will be set on ‘unstructured’ and the model which yields the lowest Akaike’s information criterion will be chosen. The following baseline measurements will be considered as covariates: age, gender, duration of pain, concomitant neck pain and history of shoulder pain.^28–30^

#### Secondary analyses

The cost effectiveness will be evaluated using the incremental cost per QALY gained of the corticosteroid injection versus physiotherapist-led exercise therapy, using both the healthcare as well as the societal perspective, using a time horizon of 12 months. Non-parametric bootstrapping will be used to depict the degree of uncertainty for costs and health effects and the cost-utility ratio in a cost-effectiveness plane. In addition, an acceptability curve will be drawn, which indicates the probability that the intervention studied has lower incremental costs per QALY gained than various thresholds for the maximum willingness to pay for an extra QALY. Similar methods will be used to estimate the cost effectiveness of both interventions in the short term (6 weeks and 3 months) and medium term (6 and 9 months).

In addition, secondary analyses include shoulder pain-intensity, shoulder function, global perceived effect, quality of life, sleep quality, work absence, healthcare utilisation and side effects and will be evaluated at all follow-up time points using linear model regression methods for numerical outcomes and logistic regression methods for dichotomous outcomes. The clinical effectiveness at all other follow-up time points will be estimated using similar methods described for the primary analyses.

#### Subgroup analysis

Two explorative, pre-defined, subgroup analyses will be performed assessing the interaction effects between treatment and the severity of baseline pain (SPADI pain subscale) and between treatment and baseline function (SPADI function subscale) on the primary and secondary outcomes.

#### Sensitivity analysis

To test the robustness of the results sensitivity analysis will be performed using per-protocol principles (excluding participants with cross-over during the study period) and using complete cases only.

### Data monitoring

This study has negligible risk according to the risk classification published in the guidelines of the Dutch Federation of University Medical Centres.^31^ Therefore, monitoring will take place once a year by independent monitors and no Data Monitoring Committee will be assigned to this study. Trial conduct and data integrity will be audited once per year by independent auditors.

### Harms

Potential adverse events will be monitored using patient self-report questionnaires, contact with the SIX research team and GP reports. GPs and physiotherapists will be asked to report any SAE and suspected unexpected serious adverse reaction (SUSAR) directly to the SIX research team. The SIX research team will report the SAE or SUSAR to the the Medical Research and Ethics Committee (METC).

### Patient and public involvement

Prior to the design of this trial, patients who recently consulted their GP for shoulder pain were contacted to participate in our patient panel. These patients could comment on the design and confirmed this study as relevant and feasible. The patient panel will also be used to help facilitate dissemination of the final results to trial participants and in the design of implementation strategies towards patients.

## Ethics and dissemination

### Ethics approval and informed consent

Ethical approval on this protocol (V.3.0) was obtained on 18 September 2020 by the METC of Erasmus MC University Medical Center Rotterdam (MEC 2020-0300). Any substantial amendment made to the protocol by the coordinating investigator is sent to the METC for approval, prior to implementation. All participants will give written informed consent prior to data collection (online supplemental file).

10.1136/bmjopen-2021-050101.supp1 Supplementary data

### Dissemination

Results of this trial will be published in peer-reviewed journals, as a double publication in a national GPs journal, to the Royal Dutch Society for Physiotherapy (KNGF), and through social media. A patient panel composed by the research team consisting of patients with shoulder pain will help facilitate the optimisation of the method of dissemination of the results to participating patients. Furthermore, participating GPs and physiotherapist will be informed about trial results (expected in 2023).

## Discussion

This paper presents the design of a pragmatic, RCT that will assess the effectiveness of corticosteroid injection versus physiotherapist-led exercise therapy for shoulder pain in primary care. Furthermore, this trial will assess the cost effectiveness of both interventions from a societal and healthcare perspective. The primary outcome is shoulder pain-intensity and function measured with the SPADI over a 12-month period. Secondary outcomes are measured at 6 weeks, 3, 6, 9 and 12 months follow-up and include shoulder pain-intensity, shoulder function, global perceived effect, quality of life, sleep quality, work absence, healthcare utilisation and adverse reactions. Between group differences for the primary outcome will be evaluated using a repeated measurements analysis with linear mixed models. An economic evaluation will be performed using a cost-utility analysis with quality of life. The outcomes of this trial may impact the clinical guideline recommendations for the management of shoulder pain in primary care and possibly the reimbursement of physiotherapy for patients with shoulder pain. Recruitment of eligible patients is currently ongoing (November 2020). Substantial protocol amendments will be communicated to participants, cooperating GPs and physiotherapist, the METC, the Dutch Trial Registry, ZonMw and the journal publishing this protocol.

## Supplementary Material

Reviewer comments

Author's manuscript
