# Supplementary material for: Clinical and cost effectiveness of a corticosteroid injection versus exercise therapy for shoulder pain in general practice: protocol for a randomised controlled trial (SIX Study)
Source: BMJ Open. 2021 Mar 30;11(3):e050101. doi: 10.1136/bmjopen-2021-050101 (PMC8011792; doi:10.1136/bmjopen-2021-050101)
Supplement: Supplementary data [file bmjopen-2021-050101supp001.pdf]

Bijlage C: toestemmingsformulier proefpersoon

De SIX Schouder Studie

- Ik heb de informatiebrief gelezen. Ook kon ik vragen stellen. Mijn vragen zijn voldoende beantwoord. Ik had genoeg tijd om te beslissen of ik meedoe.
- Ik weet dat meedoen vrijwillig is. Ook weet ik dat ik op ieder moment kan beslissen om toch niet mee te doen of te stoppen met het onderzoek. Daarvoor hoef ik geen reden te geven.
- Ik geef toestemming voor het informeren van mijn huisarts dat ik meedoe aan dit onderzoek.
- Ik geef toestemming voor het verzamelen en gebruiken van mijn gegevens voor de beantwoording van de onderzoeksvraag in dit onderzoek.
- Ik weet dat voor de controle van het onderzoek sommige mensen toegang tot al mijn gegevens kunnen krijgen. Die mensen staan vermeld in deze informatiebrief. Ik geef toestemming voor die inzage door deze personen.
- Ik geef □ **wel**

  □ **geen**

  toestemming om mijn persoonsgegevens langer te bewaren en te gebruiken voor toekomstig onderzoek op het gebied van schouderklachten.
- Ik geef □ **wel**

  □ **geen**

  toestemming om mij na dit onderzoek opnieuw te benaderen voor een vervolgonderzoek.
- Ik wil meedoen aan dit onderzoek.

Naam proefpersoon:
Handtekening: Datum : __ / __ / __

Ik verklaar dat ik deze proefpersoon volledig heb geïnformeerd over het genoemde onderzoek.

Als er tijdens het onderzoek informatie bekend wordt die de toestemming van de proefpersoon zou kunnen beïnvloeden, dan breng ik hem/haar daarvan tijdig op de hoogte.

Naam onderzoeker (of diens vertegenwoordiger):
Handtekening: Datum: __ / __ / __

----

van Doorn PF, *et al. BMJ Open* 2021; 11:e050101. doi: 10.1136/bmjopen-2021-050101
