# Supplementary material for: Clinical and cost effectiveness of a corticosteroid injection versus exercise therapy for shoulder pain in general practice: protocol for a randomised controlled trial (SIX Study)
Source: BMJ Open. 2021 Mar 30;11(3):e050101. doi: 10.1136/bmjopen-2021-050101 (PMC8011792; doi:10.1136/bmjopen-2021-050101)
Supplement: Author's manuscript [file bmjopen-2021-050101.draft_revisions.pdf]

BMJ Open

BMJ Open is committed to open peer review. As part of this commitment we make the peer review history of every article we publish publicly available.

When an article is published we post the peer reviewers' comments and the authors' responses online. We also post the versions of the paper that were used during peer review. These are the versions that the peer review comments apply to.

The versions of the paper that follow are the versions that were submitted during the peer review process. They are not the versions of record or the final published versions. They should not be cited or distributed as the published version of this manuscript.

BMJ Open is an open access journal and the full, final, typeset and author-corrected version of record of the manuscript is available on our site with no access controls, subscription charges or pay-per-view fees (http://bmjopen.bmj.com).

If you have any questions on BMJ Open's open peer review process please email info.bmjopen@bmj.com

BMJ Open

The clinical and cost effectiveness of a corticosteroid injection versus exercise therapy for shoulder pain in general practice: the SIX study a randomized clinical trial study protocol

Journal:	*BMJ Open*
Manuscript ID	bmjopen-2021-050101
Article Type:	Protocol
Date Submitted by the Author:	10-Feb-2021
Complete List of Authors:	van Doorn, Pieter; Erasmus MC, General Practice<br>de Schepper, E; Erasmus MC, General Practice<br>Schiphof, Dieuwke; Erasmus MC, General Practice<br>Ottenheijm, Ramon; Maastricht University, Family Medicine<br>Thoomes-de Graaf, Marloes; Fysio-Experts<br>Koopmanschap, M.; Erasmus University Rotterdam, Health economics and HTA<br>van Ochten, J.M.; Erasmus MC, General Practice<br>van der Windt, Danielle; Keele University, Primary Care Musculoskeletal Research Centre<br>Bindels, Patrick; Erasmus MC, General Practice<br>Koes, Bart; Erasmus MC, General Practice<br>Runhaar, Jos; Erasmus MC, General Practice
Keywords:	PRIMARY CARE, Shoulder < ORTHOPAEDIC & TRAUMA SURGERY, Clinical trials < THERAPEUTICS

SCHOLARONE™
Manuscripts

I, the Submitting Author has the right to grant and does grant on behalf of all authors of the Work (as defined in the below author licence), an exclusive licence and/or a non-exclusive licence for contributions from authors who are: i) UK Crown employees; ii) where BMJ has agreed a CC-BY licence shall apply, and/or iii) in accordance with the terms applicable for US Federal Government officers or employees acting as part of their official duties; on a worldwide, perpetual, irrevocable, royalty-free basis to BMJ Publishing Group Ltd ("BMJ") its licensees and where the relevant Journal is co-owned by BMJ to the co-owners of the Journal, to publish the Work in this journal and any other BMJ products and to exploit all rights, as set out in our licence.

The Submitting Author accepts and understands that any supply made under these terms is made by BMJ to the Submitting Author unless you are acting as an employee on behalf of your employer or a postgraduate student of an affiliated institution which is paying any applicable article publishing charge ("APC") for Open Access articles. Where the Submitting Author wishes to make the Work available on an Open Access basis (and intends to pay the relevant APC), the terms of reuse of such Open Access shall be governed by a Creative Commons licence – details of these licences and which Creative Commons licence will apply to this Work are set out in our licence referred to above.

Other than as permitted in any relevant BMJ Author's Self Archiving Policies, I confirm this Work has not been accepted for publication elsewhere, is not being considered for publication elsewhere and does not duplicate material already published. I confirm all authors consent to publication of this Work and authorise the granting of this licence.

For peer review only

The clinical and cost effectiveness of a corticosteroid injection versus exercise therapy for shoulder pain in general practice: the SIX study a randomized clinical trial study protocol

P.F. van Doorn[1*], Dr. E.I.T. de Schepper[1], Dr. D. Schiphof[1], Dr. R.P.G. Ottenheijm[2], Dr. M. Thoomes-de Graaf[3], Dr. M. Koopmanschap[4], Dr. J.M. van Ochten[1], Prof. Dr. D.A. van der Windt[5], Prof. Dr. P.J.E. Bindels[1], Prof. Dr. B.W. Koes[1], Dr. J. Runhaar[1,5],

[1] Department of General Practice, Erasmus Medical Center, University Medical Center, PO box 2040, 3000 CA Rotterdam, The Netherlands

[2] Department of Family Medicine, CAPHRI Care and Public Health Research Institute, Maastricht University, PO BOX 616, 6200 MD Maastricht, The Netherlands

[3] Fysio Experts, Rijndijk 137, 2394 AG Hazerswoude-Rijndijk

[4] Erasmus School of Health Policy & Management, Erasmus University Rotterdam, PO box 2040, 3000 CA Rotterdam, The Netherlands

[5] School of Medicine, Primary Care Centre Versus Arthritis, Keele University, ST5 5BG, Keele, United Kingdom

Correspondence
Pieter van Doorn
Erasmus MC University Medical Center Rotterdam
Department of General Practice
PO box 2040
3000 CA Rotterdam, The Netherlands
+31-(0)10-7033749
p.vandoorn@erasmusmc.nl

For peer review only

Abstract

Introduction

Shoulder pain is common and the prognosis is often unfavourable. Dutch guidelines on the treatment of shoulder pain in primary care recommend a corticosteroid injection or a referral to exercise therapy, if initial pain management fails and pain persists. However, evidence of the effectiveness of a corticosteroid injection compared to exercise therapy, especially in the long term, is limited. This trial will assess the clinical- and cost effectiveness of a corticosteroid injection compared to physiotherapist-led exercise therapy over 12 months follow-up in patients with shoulder pain in primary care.

Methods and analysis

The SIX study is a multi-centre, pragmatic randomised clinical trial in primary care. A total of 213 patients with shoulder pain, aged ≥18 years presenting in general practice will be included. Patients will be randomised (1:1) into two groups: a corticosteroid injection or 12 sessions of physiotherapist-led exercise therapy. The effect of the allocated treatment will be assessed through questionnaires at 6 weeks and after 3, 6, 9, and 12 months. The primary outcome is patient's reported shoulder pain-intensity and function, measured with the Shoulder Pain and Disability Index, over 12 months follow-up. Secondary outcomes include cost effectiveness, pain-intensity, function, health-related quality of life, sleep quality, patient's global perceived effect, work absence, healthcare utilisation and adverse events. Between group differences will be evaluated using a repeated measurements analysis with linear effects models. A cost-utility analysis will be performed to assess the cost-effectiveness using quality-adjusted life years from a medical and societal perspective.

For peer review only

Ethics and dissemination

This study was approved by the Medical Ethics Committee of Erasmus MC University Medical Center Rotterdam (MEC 2020–0300). All participants will give written informed consent prior to data collection. The results from this study will be disseminated in international journals and implemented in the primary care guidelines on shoulder pain.

Article Summary

Strengths and limitations of this study

- This is a large pragmatic randomised controlled trial that aims to evaluate two treatment options recommended by the guidelines in the management of persistent shoulder pain in general practice, a corticosteroid injection compared to exercise therapy.
- In addition to the clinical effectiveness, a cost-effectiveness analysis will be performed for both treatments.
- This study has a long follow-up period of 12 months, allowing for the analysis of the long-term (cost-)effectiveness of both treatments.
- The pragmatic nature of this trial has its drawbacks, however it will provide a true reflection of both treatments applied in current practice.

Keywords: Shoulder pain; Corticosteroid injection; Exercise therapy; Primary care; General practice; Randomized controlled trial;

Trial registration

This trial is registered in the Dutch Trial Registry (number NL8854) at 2020-08-26 (https://www.trialregister.nl/trial/8854). Issue date: 30 august 2020

For peer review only

Introduction

Background and rationale

Shoulder pain is the third most common musculoskeletal complaint in primary health care [1-3]. The estimated incidence is reported at 30.3 per 1000 person-years [3]. The prognosis for shoulder pain is often unfavourable. Only 50% of people presenting with a new episode of shoulder pain in primary care show complete recovery within six months [4]. In general, apart from pain, patients with shoulder pain report having functional limitations which can reach a level of severity whereby they preclude work-related tasks [5]. Work absence and treatment of shoulder pain generate high costs to society and healthcare [6, 7]. A recent cost-estimation study for patients with shoulder pain consulting in primary care in Sweden estimated the mean annual costs at €4,139 per patient, with sick leave accounting for more than 80% of the total costs [7].

Guidelines for the management of shoulder pain provide treatment options based on the initial diagnosis of the general practitioner (GP) and the severity of the pain [8, 9]. The recommended management options in the guidelines are focused on controlling pain and restoring or maintaining shoulder function. The recently updated primary care guideline for shoulder pain, issued by the Dutch College of General Practitioners (DCGP) in 2019, recommends a stepped-care approach. In the first step, GPs are advised to start the treatment with advice and, if necessary, prescribe analgesics. If pain persists, the GP is recommended to either prolong or adjust analgesics, administer a local corticosteroid injection in case of severe pain or refer the patient to a physiotherapist for exercise therapy in case of (impending) dysfunction [8]. Although the guideline recommends exercise therapy or corticosteroid injection when shoulder pain persists, the guideline acknowledges the lack of evidence to favour one option over the other.

For peer review only

A systematic review by Steuri et al. (2017) on RCTs comparing corticosteroid injection(s) to exercise therapy showed that injections have statistically significant, but small effect on pain in the short term, generally within 6 weeks after the intervention, but not at longer follow-up time intervals (3-6 months) [10]. Given the low quality of most of the included studies and high level of heterogeneity, the authors concluded that larger, high quality trials are required. Moreover, the authors call for health economic evaluations alongside such trials to assess comparative cost-effectiveness and cost utility. A similar call came from the Cochrane review by Page et al. (2016) on manual therapy and exercise for rotator cuff disease; 'high quality RCTs are needed to establish the benefits and harms of exercise interventions that reflect actual practice, compared with placebo, no intervention or active interventions with evidence of benefit (e.g. glucocorticoid injection)' [11].

Given the high incidence and costs associated with shoulder pain and the lack of high quality evidence to underpin current clinical practice and guideline recommendations, the recently published National Research Agenda by the NHG listed research on the effectiveness of corticosteroid injections for shoulder pain in general practice as a top priority [12]. We have therefore designed a randomised controlled trial to compare the clinical and cost-effectiveness of corticosteroid injections and physiotherapist-led exercise therapy as primary care management interventions for patients with shoulder pain.

For peer review only

Objectives

The primary objective of the Shoulder Injection and eXercise (SIX) trial is to compare the clinical effectiveness of a local corticosteroid injection to physiotherapist-led exercise therapy for shoulder pain in primary care over 12 months of follow-up. The main secondary objective is to compare the cost effectiveness of both treatments over a 12 months follow-up period.

Methods and analysis

Trial design/Study setting

The study is a randomised, multicentre, open label, parallel group, pragmatic clinical trial. Patients will be recruited in Dutch general practices. GPs will select patients presented with shoulder pain who are suitable for both a local corticosteroid injection and physiotherapist-led exercise therapy. GPs will refer these patients to the SIX research team, who will further assess all potential patients for eligibility and will undertake informed consent procedures.

Eligibility criteria

Inclusion criteria:

- Patient has contacted their GP with shoulder pain due to subacromial pain syndrome or glenohumeral disorders
- Aged 18 years or older
- Qualified for both a local corticosteroid injection and physiotherapist-led exercise therapy, as indicated by the GP
- Able to understand spoken and written Dutch language

Exclusion criteria

- Shoulder pain due to recent serious trauma, malignancy, systemic rheumatologically disease, neurological or cardiac disease) [8]

For peer review only

- Shoulder pain due to instability of the glenohumeral joint, disorders of the acromio- or sternoclavicular joint, or neck pain with additional shoulder pain
- Treatment of the affected shoulder with corticosteroid injection or physiotherapy in the last 6 months
- A history of serious shoulder trauma, such as fractures, ruptures, luxation or surgery
- Contraindications for corticosteroid injection
- Current use of oral corticosteroids

For participants with bilateral shoulder pain, the most painful shoulder will be taken as the study shoulder.

Parallel cohort study

Patients with shoulder pain who are not eligible for trial participation or patients who are eligible but do not want to be randomised, e.g. due to strong treatment preferences, will be invited to participate in a parallel cohort study. With their consent, these patients will be assessed using the same outcome measures at similar time points. In addition, these patients will complete a questionnaire regarding their treatment preferences and reasons for not wanting to participate in the trial (if applicable) at baseline. This information will provide important information regarding the recruitment process by indicating if and why recruitment may be suboptimal or failing. Furthermore, the parallel cohort study will provide the unique possibility to compare baseline characteristics of randomised participants to those who were not eligible or not willing to be randomised and outcomes following their (preferred) treatment.

We anticipate recruiting around 600 patients to this parallel cohort study. All cohort participants that are not eligible for the RCT will be informed that if the initial GP treatment fails and they consider re-consultation, they are potentially eligible for the RCT. They can contact the SIX research team for receiving additional information regarding the trial and to initiate the consent procedure for the trial.

For peer review only

Interventions

Corticosteroid injection

The corticosteroid injection will be delivered by the GP. The corticosteroid injection will consists of 40 mg triamcinolone acetonide (Kenacort-A 40), possibly in combination with a local anaesthetic agent, lidocaine 10 mg, at the discretion of the GP in accordance with the NHG guideline for shoulder pain [8].

The site of the injection, subacromial or intra-articular, will depend on the initial diagnosis of the GP. Subacromial injections will be administered to participants diagnosed with subacromial pain syndrome and intra-articular injections on participants with glenohumeral joint pain. GPs are advised to follow the instructional videos on subacromial and intra-articular injection published by the NHG [13]. All participating GPs will be invited for an optional shoulder injection training by an experienced doctor of orthopaedic medicine at the Erasmus MC.

Consultations with the GP will be coordinated so that participants typically receive their injection within one week of randomisation. In line with the guideline, a maximum of 2 injections will be permitted per patient, with the second injection, when considered necessary, offered 2 to 4 weeks after the first injection. Any participant receiving a second injection will have the date of administration recorded in their case report form.

For peer review only

Physiotherapist-led exercise therapy

Participants randomized to physiotherapist-led exercise therapy will be referred to one of the local physiotherapists. Preferably the physiotherapist is affiliated with the Dutch Shoulder Network (SNN). The SNN is an umbrella organisation for regional shoulder networks of physiotherapist practices. All affiliated physiotherapists have to complete a 2-day entry course on shoulder pain, accredited by the Royal Dutch Society for Physiotherapy (KNGF).

The exercise therapy will consist of a maximum of 12 treatments of around 30 minutes under the supervision of the physiotherapist over a course of 12-14 weeks. In addition, all participants will receive home-based exercise at the discretion of the physiotherapist. The intensity of the exercise is based on tissue irritability and the capacity of the patient. Pain during or after exercise is allowed, as long as there is no night-time pain and the pain returns to pre-training levels within 24 hours. Physiotherapist will be requested not to use massage, laser therapy, ultrasound therapy, transcutaneous electrical nerve stimulation (TENS), dry needling or acupuncture, given lack of evidence for effectiveness [14]. All participating physiotherapists will receive a brief guideline developed in cooperation with the SNN describing the criteria for exercise therapy.

C*o-interventions*

This is a pragmatic clinical trial designed to evaluate the effectiveness of corticosteroid injections compared to physiotherapist-led exercise therapy for shoulder pain in real-life routine practice conditions. Therefore, participants will be instructed to continue their usual medication as discussed with their GP. Co-interventions after randomisation will be allowed and will be monitored through medical record review and questionnaires. This includes cross-over between interventions, which is estimated to occur in 20% of participants based on the number of patients receiving an injection and referral for exercise therapy in the 'usual care' treatment arm of a recent RCT [15].

For peer review only

Outcomes

The selection of outcome measures has been based on the core outcome set published by OMERACT [16]. The primary outcome is shoulder pain-intensity and function measured using the Shoulder Pain and Disability Index (SPADI) total score over 12 months post randomisation [17]. The SPADI is the most commonly used measure to assess pain-intensity and disability [18]. The Dutch version of the SPADI has good psychometric properties [19, 20].

Secondary outcomes include incremental costs per quality adjusted life year (QALY) gained, using both the medical as well as the societal perspective, over 12 months post randomisation. Medical costs will be measured using the Medical Cost Questionnaire (MCQ) and societal costs will be measured using the Productivity Cost Questionnaire (PCQ) [21]. QALY will be measured using the five-level version of the well-validated EuroQol Five-Dimensional Questionnaire (EQ-5D-5L) score [22].

Other secondary outcomes will be clinical- and cost effectiveness of the randomised treatments in the short term (6 weeks, 3 months) and medium term (6 months, 9 months). In addition, secondary outcomes will include subdomains (pain and function) of the SPADI, health-related quality of life (EQ-5D-5L), sleep quality measured with the Sleep Quality Scale (SQS) [23], participant's perceived recovery using the global perceived effect questionnaire [16], work absence as measured by the PCQ , healthcare utilisation as measured by the MCQ, side effects assessed at short term post randomisation and serious adverse events (SAE) occurring post randomisation.

For peer review only

Participant timeline

Time-point	Pre randomisation	Baseline (T0)	6 weeks (T1)	3 months (T2)	6-9-12 months (T3-T4-T5)[a]
**Enrolment**					
Diagnosis	X				
Eligibility screening	X				
Informed consent	X				
Randomisation[b]		X			
**Interventions**					
Corticosteroid injection[b]		X			
Physiotherapist-led exercise therapy[b]		←	—	→	
**Assessments**					
Socio-demographics		X			
Current shoulder episode (location, duration, cause, course, stiffness)		X			
Previous shoulder episodes (history, treatments)		X			
Other current pain locations (pain manikin)		X			
Other relevant medical issues		X			
Psychological prognostic factors (HADS, FABQ)		X			
Current medical use for the shoulder pain		X			
Treatment preferences		X			
Treatment expectations		X			
**Outcomes**					
Pain and function (SPADI)		X	X	X	X
Medical costs (MCQ)			X	X	X
Global perceived effect (GPE)			X	X	X
Productivity costs (PCQ)			X	X	X
Health-related quality of life (EQ-5D-5L)		X	X	X	X
Sleep quality (SQS)		X	X	X	X
Side effects			X	X	
Serious adverse events (SAE)			X	X	X

[a]At these time-points the indicated outcome measures will be repeated. [b]Randomisation occurs after baseline measurements are taken.

For peer review only

Sample size

The target sample size is 85 participants in each trial group. This is based on 90% power and a 0.05 two-sided statistical significance to detect a minimally clinically important difference of ten points on the SPADI total scale [24], using a conservative estimation of a baseline SD of 20 [25]. Accounting for a potential loss to follow-up at 12 months of 20%, this will require a total of 213 patients to be randomized to the intervention groups.

Recruitment

All patients (≥18 years old) consulting their GP for shoulder pain who are suitable for both a local corticosteroid injection and physiotherapist-led exercise therapy can be invited by their GP to participate in this study. These patients will be informed on the trial by the GP and are advised to contact the research team. The research team will provide further information on the trial and if the patient confirms their interest to participate in the trial, eligibility will be checked and the informed consent procedure will be completed. After the participant has completed the baseline questionnaire, the patient will be randomised by the research team. The patient and the GP will be notified on the randomisation result by the research ream.

All other patients (e.g., wait-and see policy or prescription of analgesics) will be invited to participate in the parallel cohort study. These patients will be invited through two-weekly searches of the medical records of participating GP practices. All cohort participants will be informed that if the initial GP treatment fails and they consider re-consultation, they are potentially eligible for the RCT (figure 1).

Accounting for a 25% willingness of patients to participate in the RCT and in the cohort, a 50% willingness of participants in the cohort to enter the trial if initial treatment fails, and a 25% loss due to not eligible for the trial, 2430 patients need to be invited to participate in either the trial or the cohort

For peer review only

over the 18 months period. On average a fulltime Dutch GP has around 2095 registered patients [26]. With an incidence rate of 30.3 per 1000 person-years in the Netherlands [3], it is expected that a fulltime GP sees around 6 patients with a new episode of shoulder pain per month, which results in 23 GPs needed for this study. However, taking in account Lasagna's law [27], we expect to need at least 46 GPs to ensure the total sample size.

Allocation

The Erasmus MC Clinical Trial Center, who will not meet or contact the patients will prepare a remote web-based randomization system using random blocks of 8, 6 or 4 to ensure concealment of allocation. Participants and their GP will be informed about the outcome of the randomisation by phone and participants will receive a patient card through mail detailing their treatment allocation and related procedures.

Blinding

The researcher who will carry out the primary analysis will be blinded for treatment allocation. The participant and the GP will not be blinded for treatment allocation. This is not feasible in this pragmatic trial.

Data collection methods

After obtaining informed consent, participants will complete the baseline questionnaire. Subsequently, the participants will be randomized to one of the two interventions groups. Participants will be asked to complete online questionnaires sent by e-mail, after 6 weeks, 3, 6, 9 and 12 months after randomisation. If the follow-up questionnaire is not returned within 2 weeks of initial mailing, a reminder will be sent encouraging the participant to complete the questionnaire. Non-responders or

For peer review only

responders with incomplete questionnaires will be contacted by telephone to pose them the missing questions.

Data management

Data management will be performed via a web-based medical survey tracker (Gemstracker). Each participant will be allocated a unique code, which will be used on all trial-specific documents, except for the signed informed consent and contact details. Participants' identifiable data will be stored separately and securely from study data in accordance with local procedures.

Statistical methods

Baseline characteristics will be summarized using descriptive statistics. All analyses will be performed under intention-to-treat (ITT).

Primary analyses

The primary clinical outcome is patient reported severity of pain and function over 12 months post-randomisation, measured with the SPADI total score. A linear mixed model with repeated measures will be used to generate estimates of effects. The time-points included in this model will be baseline, 6 weeks, 3, 6, 9, and 12 months. Baseline values for the primary outcome are retained as part of the outcome vector and group means on the primary outcome are assumed to be equal at baseline (i.e. an intervention-effect is restricted at baseline). Fixed effects will be time and time by treatment group. To model the covariance of repeated measures by participant, the option for data structure in the analyses will be set on 'unstructured' and the model which yields the lowest Akaike's information criterion will be chosen. The following baseline measurements will be considered as covariates: age, gender, duration of pain, concomitant neck pain and history of shoulder pain [28-30].

For peer review only

Secondary analyses

The cost effectiveness will be evaluated using the incremental cost per QALY gained of the corticosteroid injection versus physiotherapist-led exercise therapy, using both the healthcare as well as the societal perspective, using a time horizon of 12 months. Non-parametric bootstrapping will be used to depict the degree of uncertainty for costs and health effects and the cost-utility ratio in a cost-effectiveness plane. In addition, an acceptability curve will be drawn, which indicates the probability that the intervention studied has lower incremental costs per QALY gained than various thresholds for the maximum willingness to pay for an extra QALY. Similar methods will be used to estimate the cost effectiveness of both interventions in the short term (6 weeks and 3 months) and medium term (6 and 9 months).

In addition, secondary analyses include shoulder pain-intensity, shoulder function, global perceived effect, quality of life, sleep quality, work absence, healthcare utilisation and side effects and will be evaluated at all follow up time points using linear model regression methods for numerical outcomes and logistic regression methods for dichotomous outcomes. The clinical effectiveness at all other follow-up time points will be estimated using similar methods described for the primary analyses.

Subgroup analysis

Two explorative, pre-defined, subgroup analyses will be performed assessing the interaction effects between treatment and the severity of baseline pain (SPADI pain subscale) and between treatment and baseline function (SPADI function subscale) on the primary and secondary outcomes.

Sensitivity analysis

To test the robustness of the results sensitivity analysis will be performed using per-protocol principles (excluding participants with cross-over during the study period) and using complete cases only.

For peer review only - http://bmjopen.bmj.com/site/about/guidelines.xhtml

Data monitoring

This study has negligible risk according to the risk classification published in the guidelines of the Dutch Federation of University Medical Centres (NFU) [31]. Therefore, monitoring will take place once a year by independent monitors and no Data Monitoring Committee (DMC) will be assigned to this study. Trial conduct and data integrity will be audited once per year by independent auditors.

Harms

Potential adverse events will be monitored using patient self-report questionnaires, contact with the SIX research team and general practitioner reports. GPs and physiotherapists will be asked to report any serious adverse event (SAE) and suspected unexpected serious adverse reaction (SUSAR) directly to the SIX research team. The SIX research team will report the SAE or SUSAR to the the Medical Research and Ethics Committee (METC).

Patient and public involvement

Prior to the design of this trial, patients who recently consulted their GP for shoulder pain were contacted to participate in our patient panel. These patients could comment on the design and confirmed this study as relevant and feasible. The patient panel will also be used to help facilitate dissemination of the final results to trial participants and in the design of implementation strategies towards patients.

For peer review only

Ethics and Dissemination

Ethics approval and informed consent

Ethical approval was obtained by the Medical Ethics Committee (METC) of Erasmus MC University Medical Center Rotterdam (MEC 2020–0300). Any substantial amendment made to the protocol by the coordinating investigator is sent to the METC for approval, prior to implementation. All participants will give written informed consent prior to data collection.

Dissemination

Results of this trial will be published in peer-reviewed journals, as a double publication in a national general practitioners journal, to the Royal Dutch Society for Physiotherapy (KNGF), and through social media. A patient panel composed by the research team consisting of patients with shoulder pain will help facilitate the optimization of the method of dissemination of the results to participating patients. Furthermore, participating GPs and physiotherapist will be informed about trial results (expected in 2023).

Discussion

This paper presents the design of a pragmatic, randomized controlled trial that will assess the effectiveness of corticosteroid injection versus physiotherapist-led exercise therapy for shoulder pain in primary care. Furthermore, this trial will assess the cost effectiveness of both interventions from a societal and healthcare perspective. The primary outcome is shoulder pain-intensity and function measured with the SPADI over a 12 months period. Secondary outcomes are measured at 6 weeks, 3, 6, 9 and 12 months follow-up and include shoulder pain-intensity, shoulder function, global perceived effect, quality of life, sleep quality, work absence, healthcare utilisation and adverse reactions. Between

For peer review only

group differences for the primary outcome will be evaluated using a repeated measurements analysis with linear mixed models. An economic evaluation will be performed using a cost utility analysis with quality of life. The outcomes of this trial may impact the clinical guideline recommendations for the management of shoulder pain in primary care and possibly the reimbursement of physiotherapy for patients with shoulder pain. Recruitment of eligible patients is currently ongoing (November 2020). Substantial protocol amendments will be communicated to participants, cooperating GPs and physiotherapist, the Medical Research and Ethics Committee (METC), the Dutch Trial Registry, ZonMw and the journal publishing this protocol.

Acknowledgements

The authors would like to thank Gerard Koel for his contributions in the design of the exercise therapy guideline for participating physiotherapists.

Statements

Contributorship statement

JR, BK, PB, EdS, MK, RO, DS, MTdG, DvdW, JvO participated in the design of the study. EdS, PvD, JR and BK coordinate the trial and are responsible for data collection. PvD and JR prepared the article. All authors have read and approved the final manuscript.

Competing interests

The authors declare that they have no conflict of interest.

For peer review only

Funding

This trial is funded by the Netherlands Organization for Health Research and Development (ZonMw),

Grant number: 852002027

Data sharing statement

Not applicable.

For peer review only

References

1. Bot SD, van der Waal JM, Terwee CB, van der Windt DA, Schellevis FG, Bouter LM, Dekker J: **Incidence and prevalence of complaints of the neck and upper extremity in general practice**. *Ann Rheum Dis* 2005, **64**(1):118-123.
2. Greving K, Dorrestijn O, Winters JC, Groenhof F, van der Meer K, Stevens M, Diercks RL: **Incidence, prevalence, and consultation rates of shoulder complaints in general practice**. *Scand J Rheumatol* 2012, **41**(2):150-155.
3. van Doorn PF, de Schepper EIT, Rozendaal RM, Ottenheijm RPG, van der Lei J, Bindels PJ, Schiphof D: **The incidence and management of shoulder complaints in primary care: a retrospective cohort study**. *Fam Pract* 2020, **Manuscript submitted for publication**.
4. Kuijpers T, van der Windt DA, van der Heijden GJ, Bouter LM: **Systematic review of prognostic cohort studies on shoulder disorders**. *Pain* 2004, **109**(3):420-431.
5. Smith-Forbes EV, Moore-Reed SD, Westgate PM, Kibler WB, Uhl TL: **Descriptive analysis of common functional limitations identified by patients with shoulder pain**. *J Sport Rehabil* 2015, **24**(2):179-188.
6. Kuijpers T, van Tulder MW, van der Heijden GJ, Bouter LM, van der Windt DA: **Costs of shoulder pain in primary care consulters: a prospective cohort study in The Netherlands**. *BMC Musculoskelet Disord* 2006, **7**:83.
7. Virta L, Joranger P, Brox JI, Eriksson R: **Costs of shoulder pain and resource use in primary health care: a cost-of-illness study in Sweden**. *BMC Musculoskelet Disord* 2012, **13**:17.
8. **NHG Standaard Schouderklachten** [https://richtlijnen.nhg.org/standaarden/schouderklachten]
9. Codsi M, Howe CR: **Shoulder Conditions: Diagnosis and Treatment Guideline**. *Phys Med Rehabil Clin N Am* 2015, **26**(3):467-489.
10. Steuri R, Sattelmayer M, Elsig S, Kolly C, Tal A, Taeymans J, Hilfiker R: **Effectiveness of conservative interventions including exercise, manual therapy and medical management in adults with shoulder impingement: a systematic review and meta-analysis of RCTs**. *Br J Sports Med* 2017, **51**(18):1340-1347.
11. Page MJ, Green S, McBain B, Surace SJ, Deitch J, Lyttle N, Mrocki MA, Buchbinder R: **Manual therapy and exercise for rotator cuff disease**. *Cochrane Database Syst Rev* 2016(6):CD012224.
12. Nederlandse Huisartsen Genootschap: **Nationale Onderzoeksagenda Huisartsgeneeskunde**. In. www.nhg.org: NHG; 2018.
13. Nederlands Huisartsen Genootschap (NHG): **Subacromiale/Intra-articulaire injectie van de schouder**. In. portal.nhg.org; 2012.
14. Jansen MJ, Brooijmans F, Geraets JJXR, Lenssen AF, R.P.G. O, Penning LIF, de Bie RA: **KNGF evidence statement Subacromiale klachten**. In., vol. 121. www.kngfrichtlijnen.nl: Koninklijk Genootschap voor Fysiotherapie (KNGF); 2011.
15. Ottenheijm RP, Cals JW, Winkens B, Weijers RE, de Bie RA, Dinant GJ: **Ultrasound imaging to tailor the treatment of acute shoulder pain: a randomised controlled trial in general practice**. *BMJ Open* 2016, **6**(11):e011048.
16. Ramiro S, Page MJ, Whittle SL, Huang H, Verhagen AP, Beaton DE, Richards P, Voshaar M, Shea B, van der Windt DA *et al*: **The OMERACT Core Domain Set for Clinical Trials of Shoulder Disorders**. *J Rheumatol* 2019, **46**(8):969-975.
17. Roach KE, Budiman-Mak E, Songsiridej N, Lertratanakul Y: **Development of a shoulder pain and disability index**. *Arthritis Care Res* 1991, **4**(4):143-149.

For peer review only

18. Page MJ, McKenzie JE, Green SE, Beaton DE, Jain NB, Lenza M, Verhagen AP, Surace S, Deitch J, Buchbinder R: **Core domain and outcome measurement sets for shoulder pain trials are needed: systematic review of physical therapy trials**. *J Clin Epidemiol* 2015, **68**(11):1270-1281.

19. Thoomes-de Graaf M, Scholten-Peeters GG, Duijn E, Karel Y, Koes BW, Verhagen AP: **The Dutch Shoulder Pain and Disability Index (SPADI): a reliability and validation study**. *Qual Life Res* 2015, **24**(6):1515-1519.

20. Thoomes-de Graaf M, Scholten-Peeters W, Duijn E, Karel Y, de Vet HC, Koes B, Verhagen A: **The Responsiveness and Interpretability of the Shoulder Pain and Disability Index**. *J Orthop Sports Phys Ther* 2017, **47**(4):278-286.

21. Bouwmans C, Krol M, Severens H, Koopmanschap M, Brouwer W, Hakkaart-van Roijen L: **The iMTA Productivity Cost Questionnaire: A Standardized Instrument for Measuring and Valuing Health-Related Productivity Losses**. *Value Health* 2015, **18**(6):753-758.

22. M MV, K MV, S MAAE, de Wit GA, Prenger R, E AS: **Dutch Tariff for the Five-Level Version of EQ-5D**. *Value Health* 2016, **19**(4):343-352.

23. Snyder E, Cai B, DeMuro C, Morrison MF, Ball W: **A New Single-Item Sleep Quality Scale: Results of Psychometric Evaluation in Patients With Chronic Primary Insomnia and Depression**. *J Clin Sleep Med* 2018, **14**(11):1849-1857.

24. Breckenridge JD, McAuley JH: **Shoulder Pain and Disability Index (SPADI)**. *J Physiother* 2011, **57**(3):197.

25. Roddy E, Zwierska I, Hay EM, Jowett S, Lewis M, Stevenson K, van der Windt D, Foster NE, team St: **Subacromial impingement syndrome and pain: protocol for a randomised controlled trial of exercise and corticosteroid injection (the SUPPORT trial)**. *BMC Musculoskelet Disord* 2014, **15**:81.

26. Nederlandse Zorgautoriteit: **Beleidsregel huisartsenzorg en multidisciplinaire zorg**. In., 1 edn. puc.overheid.nl; 2020.

27. van der Wouden JC, Blankenstein AH, Huibers MJ, van der Windt DA, Stalman WA, Verhagen AP: **Survey among 78 studies showed that Lasagna's law holds in Dutch primary care research**. *J Clin Epidemiol* 2007, **60**(8):819-824.

28. Bot SD, van der Waal JM, Terwee CB, van der Windt DA, Scholten RJ, Bouter LM, Dekker J: **Predictors of outcome in neck and shoulder symptoms: a cohort study in general practice**. *Spine (Phila Pa 1976)* 2005, **30**(16):E459-470.

29. Engebretsen K, Grotle M, Bautz-Holter E, Ekeberg OM, Brox JI: **Predictors of shoulder pain and disability index (SPADI) and work status after 1 year in patients with subacromial shoulder pain**. *BMC Musculoskelet Disord* 2010, **11**:218.

30. Kooijman MK, Barten DJ, Swinkels IC, Kuijpers T, de Bakker D, Koes BW, Veenhof C: **Pain intensity, neck pain and longer duration of complaints predict poorer outcome in patients with shoulder pain--a systematic review**. *BMC Musculoskelet Disord* 2015, **16**:288.

31. Nederlandse Federatie van Universitair Medische Centra (NFU): **Richtlijn Kwaliteitsborging Mensgebonden Onderzoek 2019**. In.: Nederlandse Federatie van Universitair Medische Centra (NFU); March 2019.

For peer review only

Figures

Figure 1. Consort flowchart of recruitment

For peer review only

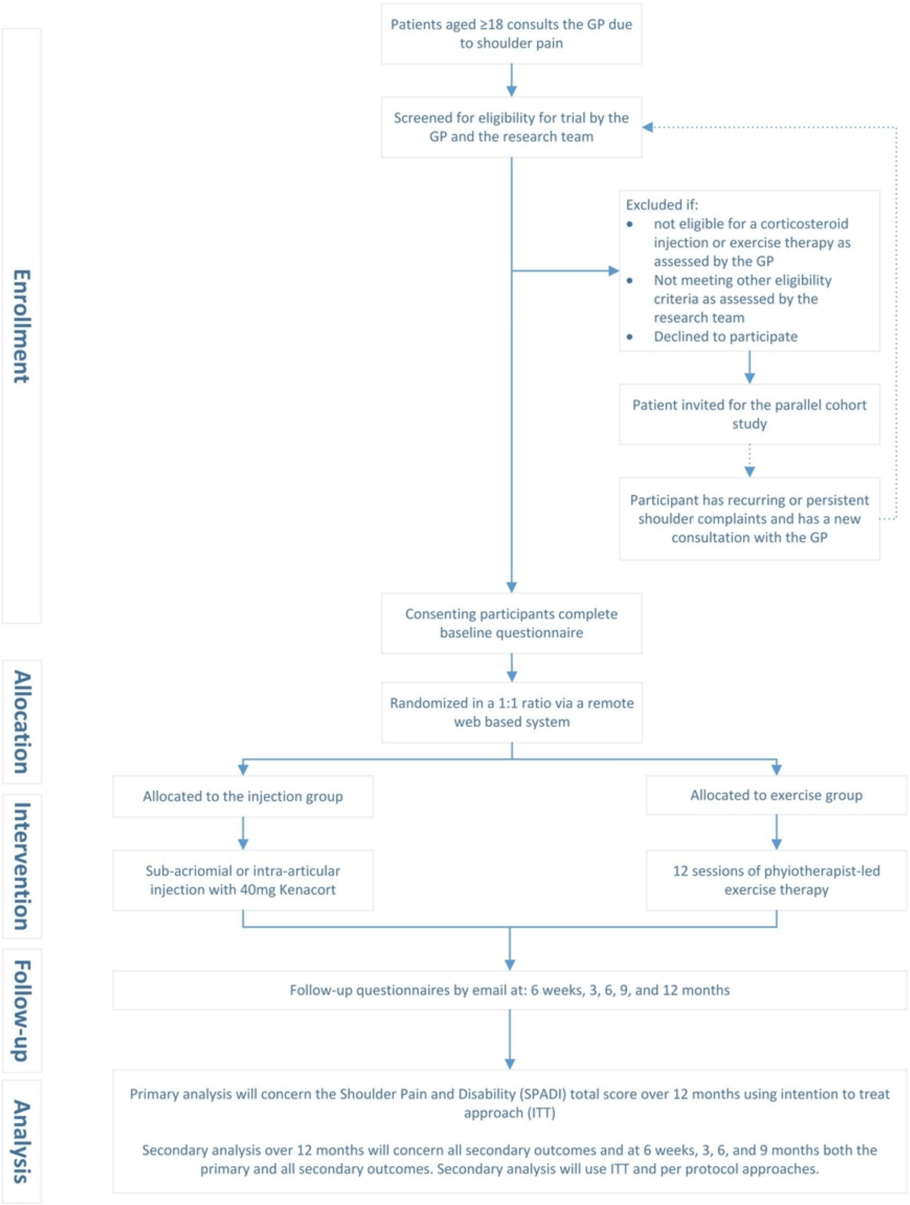

Figure 1. Consort flowchart for recruitment

36x48mm (600 x 600 DPI)

Reporting checklist for protocol of a clinical trial.

Based on the SPIRIT guidelines.

Instructions to authors

Complete this checklist by entering the page numbers from your manuscript where readers will find each of the items listed below.

Your article may not currently address all the items on the checklist. Please modify your text to include the missing information. If you are certain that an item does not apply, please write "n/a" and provide a short explanation.

Upload your completed checklist as an extra file when you submit to a journal.

In your methods section, say that you used the SPIRITreporting guidelines, and cite them as:

Chan A-W, Tetzlaff JM, Gøtzsche PC, Altman DG, Mann H, Berlin J, Dickersin K, Hróbjartsson A, Schulz KF, Parulekar WR, Krleža-Jerić K, Laupacis A, Moher D. SPIRIT 2013 Explanation and Elaboration: Guidance for protocols of clinical trials. BMJ. 2013;346:e7586

		Reporting Item	Page Number
**Administrative information**			
Title	#1	Descriptive title identifying the study design, population, interventions, and, if applicable, trial acronym	1
Trial registration	#2a	Trial identifier and registry name. If not yet registered, name of intended registry	2
Trial registration: data set	#2b	All items from the World Health Organization Trial Registration Data Set	4
Protocol version	#3	Date and version identifier	n/a
Funding	#4	Sources and types of financial, material, and other support	22
Roles and responsibilities: contributorship	#5a	Names, affiliations, and roles of protocol contributors	22

For peer review only

Roles and responsibilities: sponsor contact information	#5b	Name and contact information for the trial sponsor	22
Roles and responsibilities: sponsor and funder	#5c	Role of study sponsor and funders, if any, in study design; collection, management, analysis, and interpretation of data; writing of the report; and the decision to submit the report for publication, including whether they will have ultimate authority over any of these activities	22
Roles and responsibilities: committees	#5d	Composition, roles, and responsibilities of the coordinating centre, steering committee, endpoint adjudication committee, data management team, and other individuals or groups overseeing the trial, if applicable (see Item 21a for data monitoring committee)	22
**Introduction**			
Background and rationale	#6a	Description of research question and justification for undertaking the trial, including summary of relevant studies (published and unpublished) examining benefits and harms for each intervention	4
Background and rationale: choice of comparators	#6b	Explanation for choice of comparators	4-5
Objectives	#7	Specific objectives or hypotheses	6
Trial design	#8	Description of trial design including type of trial (eg, parallel group, crossover, factorial, single group), allocation ratio, and framework (eg, superiority, equivalence, non-inferiority, exploratory)	5
**Methods: Participants, interventions, and outcomes**			
Study setting	#9	Description of study settings (eg, community clinic, academic hospital) and list of countries where data will be collected. Reference to where list of study sites can be obtained	6
Eligibility criteria	#10	Inclusion and exclusion criteria for participants. If applicable, eligibility criteria for study centres and individuals who will	6-7

For peer review only

		perform the interventions (eg, surgeons, psychotherapists)	
Interventions: description	#11a	Interventions for each group with sufficient detail to allow replication, including how and when they will be administered	8-9
Interventions: modifications	#11b	Criteria for discontinuing or modifying allocated interventions for a given trial participant (eg, drug dose change in response to harms, participant request, or improving / worsening disease)	9
Interventions: adherance	#11c	Strategies to improve adherence to intervention protocols, and any procedures for monitoring adherence (eg, drug tablet return; laboratory tests)	9
Interventions: concomitant care	#11d	Relevant concomitant care and interventions that are permitted or prohibited during the trial	9
Outcomes	#12	Primary, secondary, and other outcomes, including the specific measurement variable (eg, systolic blood pressure), analysis metric (eg, change from baseline, final value, time to event), method of aggregation (eg, median, proportion), and time point for each outcome. Explanation of the clinical relevance of chosen efficacy and harm outcomes is strongly recommended	10
Participant timeline	#13	Time schedule of enrolment, interventions (including any run-ins and washouts), assessments, and visits for participants. A schematic diagram is highly recommended (see Figure)	11
Sample size	#14	Estimated number of participants needed to achieve study objectives and how it was determined, including clinical and statistical assumptions supporting any sample size calculations	12
Recruitment	#15	Strategies for achieving adequate participant enrolment to reach target sample size	12-13
**Methods: Assignment of interventions (for controlled trials)**			
Allocation: sequence generation	#16a	Method of generating the allocation sequence (eg, computer-generated random numbers), and list of any factors for stratification. To reduce predictability of a random sequence, details of any planned restriction (eg, blocking) should be provided in a separate document that is unavailable to those who enrol participants or assign interventions	14

For peer review only

Allocation concealment mechanism	#16b	Mechanism of implementing the allocation sequence (eg, central telephone; sequentially numbered, opaque, sealed envelopes), describing any steps to conceal the sequence until interventions are assigned	14
Allocation: implementation	#16c	Who will generate the allocation sequence, who will enrol participants, and who will assign participants to interventions	14
Blinding (masking)	#17a	Who will be blinded after assignment to interventions (eg, trial participants, care providers, outcome assessors, data analysts), and how	14
Blinding (masking): emergency unblinding	#17b	If blinded, circumstances under which unblinding is permissible, and procedure for revealing a participant's allocated intervention during the trial	14
**Methods: Data collection, management, and analysis**			
Data collection plan	#18a	Plans for assessment and collection of outcome, baseline, and other trial data, including any related processes to promote data quality (eg, duplicate measurements, training of assessors) and a description of study instruments (eg, questionnaires, laboratory tests) along with their reliability and validity, if known. Reference to where data collection forms can be found, if not in the protocol	15
Data collection plan: retention	#18b	Plans to promote participant retention and complete follow-up, including list of any outcome data to be collected for participants who discontinue or deviate from intervention protocols	15
Data management	#19	Plans for data entry, coding, security, and storage, including any related processes to promote data quality (eg, double data entry; range checks for data values). Reference to where details of data management procedures can be found, if not in the protocol	15
Statistics: outcomes	#20a	Statistical methods for analysing primary and secondary outcomes. Reference to where other details of the statistical analysis plan can be found, if not in the protocol	15-16
Statistics: additional analyses	#20b	Methods for any additional analyses (eg, subgroup and adjusted analyses)	16-17

For peer review only

Statistics: analysis population and missing data	#20c	Definition of analysis population relating to protocol non-adherence (eg, as randomised analysis), and any statistical methods to handle missing data (eg, multiple imputation)	17
**Methods: Monitoring**			
Data monitoring: formal committee	#21a	Composition of data monitoring committee (DMC); summary of its role and reporting structure; statement of whether it is independent from the sponsor and competing interests; and reference to where further details about its charter can be found, if not in the protocol. Alternatively, an explanation of why a DMC is not needed	17
Data monitoring: interim analysis	#21b	Description of any interim analyses and stopping guidelines, including who will have access to these interim results and make the final decision to terminate the trial	17
Harms	#22	Plans for collecting, assessing, reporting, and managing solicited and spontaneously reported adverse events and other unintended effects of trial interventions or trial conduct	17
Auditing	#23	Frequency and procedures for auditing trial conduct, if any, and whether the process will be independent from investigators and the sponsor	17
**Ethics and dissemination**			
Research ethics approval	#24	Plans for seeking research ethics committee / institutional review board (REC / IRB) approval	18
Protocol amendments	#25	Plans for communicating important protocol modifications (eg, changes to eligibility criteria, outcomes, analyses) to relevant parties (eg, investigators, REC / IRBs, trial participants, trial registries, journals, regulators)	18
Consent or assent	#26a	Who will obtain informed consent or assent from potential trial participants or authorised surrogates, and how (see Item 32)	18
Consent or assent: ancillary studies	#26b	Additional consent provisions for collection and use of participant data and biological specimens in ancillary studies, if applicable	n/a
Confidentiality	#27	How personal information about potential and enrolled participants will be collected, shared, and maintained in order to protect confidentiality before, during, and after the trial	18

For peer review only

Declaration of interests	#28	Financial and other competing interests for principal investigators for the overall trial and each study site	22
Data access	#29	Statement of who will have access to the final trial dataset, and disclosure of contractual agreements that limit such access for investigators	22
Ancillary and post trial care	#30	Provisions, if any, for ancillary and post-trial care, and for compensation to those who suffer harm from trial participation	n/a
Dissemination policy: trial results	#31a	Plans for investigators and sponsor to communicate trial results to participants, healthcare professionals, the public, and other relevant groups (eg, via publication, reporting in results databases, or other data sharing arrangements), including any publication restrictions	18
Dissemination policy: authorship	#31b	Authorship eligibility guidelines and any intended use of professional writers	n/a
Dissemination policy: reproducible research	#31c	Plans, if any, for granting public access to the full protocol, participant-level dataset, and statistical code	n/a
**Appendices**			
Informed consent materials	#32	Model consent form and other related documentation given to participants and authorised surrogates	n/a
Biological specimens	#33	Plans for collection, laboratory evaluation, and storage of biological specimens for genetic or molecular analysis in the current trial and for future use in ancillary studies, if applicable	n/a

The SPIRIT checklist is distributed under the terms of the Creative Commons Attribution License CC-BY-ND 3.0. This checklist was completed on 05. February 2021 using https://www.goodreports.org/, a tool made by the EQUATOR Network in collaboration with Penelope.ai

For peer review only

BMJ Open

The clinical and cost effectiveness of a corticosteroid injection versus exercise therapy for shoulder pain in general practice: Protocol for a randomized controlled trial (SIX study)

Journal:	*BMJ Open*
Manuscript ID	bmjopen-2021-050101.R1
Article Type:	Protocol
Date Submitted by the Author:	03-Mar-2021

Complete List of Authors:	van Doorn, Pieter; Erasmus MC, General Practice<br>de Schepper, E; Erasmus MC, General Practice<br>Schiphof, Dieuwke; Erasmus MC, General Practice<br>Ottenheijm, Ramon; Maastricht University, Family Medicine<br>Thoomes-de Graaf, Marloes; Fysio-Experts<br>Koopmanschap, M.; Erasmus University Rotterdam, Health economics and HTA<br>van Ochten, J.M.; Erasmus MC, General Practice<br>van der Windt, Danielle; Keele University, Primary Care Musculoskeletal Research Centre<br>Bindels, Patrick; Erasmus MC, General Practice<br>Koes, Bart; Erasmus MC, General Practice<br>Runhaar, Jos; Erasmus MC, General Practice

**Primary Subject Heading**:	General practice / Family practice
Secondary Subject Heading:	Medical management
Keywords:	PRIMARY CARE, Shoulder < ORTHOPAEDIC & TRAUMA SURGERY, Clinical trials < THERAPEUTICS

SCHOLARONE™ Manuscripts

I, the Submitting Author has the right to grant and does grant on behalf of all authors of the Work (as defined in the below author licence), an exclusive licence and/or a non-exclusive licence for contributions from authors who are: i) UK Crown employees; ii) where BMJ has agreed a CC-BY licence shall apply, and/or iii) in accordance with the terms applicable for US Federal Government officers or employees acting as part of their official duties; on a worldwide, perpetual, irrevocable, royalty-free basis to BMJ Publishing Group Ltd ("BMJ") its licensees and where the relevant Journal is co-owned by BMJ to the co-owners of the Journal, to publish the Work in this journal and any other BMJ products and to exploit all rights, as set out in our licence.

The Submitting Author accepts and understands that any supply made under these terms is made by BMJ to the Submitting Author unless you are acting as an employee on behalf of your employer or a postgraduate student of an affiliated institution which is paying any applicable article publishing charge ("APC") for Open Access articles. Where the Submitting Author wishes to make the Work available on an Open Access basis (and intends to pay the relevant APC), the terms of reuse of such Open Access shall be governed by a Creative Commons licence – details of these licences and which Creative Commons licence will apply to this Work are set out in our licence referred to above.

Other than as permitted in any relevant BMJ Author's Self Archiving Policies, I confirm this Work has not been accepted for publication elsewhere, is not being considered for publication elsewhere and does not duplicate material already published. I confirm all authors consent to publication of this Work and authorise the granting of this licence.

For peer review only

The clinical and cost effectiveness of a corticosteroid injection versus exercise therapy for shoulder pain in general practice: Protocol for a randomized controlled trial (SIX study)

P.F. van Doorn[1*], Dr. E.I.T. de Schepper[1], Dr. D. Schiphof[1], Dr. R.P.G. Ottenheijm[2], Dr. M. Thoomes-de Graaf[3], Dr. M. Koopmanschap[4], Dr. J.M. van Ochten[1], Prof. Dr. D.A. van der Windt[5], Prof. Dr. P.J.E. Bindels[1], Prof. Dr. B.W. Koes[1], Dr. J. Runhaar[1,5],

[1] Department of General Practice, Erasmus Medical Center, University Medical Center, PO box 2040, 3000 CA Rotterdam, The Netherlands

[2] Department of Family Medicine, CAPHRI Care and Public Health Research Institute, Maastricht University, PO BOX 616, 6200 MD Maastricht, The Netherlands

[3] Fysio Experts, Rijndijk 137, 2394 AG Hazerswoude-Rijndijk

[4] Erasmus School of Health Policy & Management, Erasmus University Rotterdam, PO box 2040, 3000 CA Rotterdam, The Netherlands

[5] School of Medicine, Primary Care Centre Versus Arthritis, Keele University, ST5 5BG, Keele, United Kingdom

Correspondence
Pieter van Doorn
Erasmus MC University Medical Center Rotterdam
Department of General Practice
PO box 2040
3000 CA Rotterdam, The Netherlands
+31-(0)10-7033749
p.vandoorn@erasmusmc.nl

For peer review only

Abstract

Introduction

Shoulder pain is common and the prognosis is often unfavourable. Dutch guidelines on the treatment of shoulder pain in primary care recommend a corticosteroid injection or a referral to exercise therapy, if initial pain management fails and pain persists. However, evidence of the effectiveness of a corticosteroid injection compared to exercise therapy, especially in the long term, is limited. This trial will assess the clinical- and cost effectiveness of a corticosteroid injection compared to physiotherapist-led exercise therapy over 12 months follow-up in patients with shoulder pain in primary care.

Methods and analysis

The SIX study is a multi-centre, pragmatic randomised clinical trial in primary care. A total of 213 patients with shoulder pain, aged ≥18 years presenting in general practice will be included. Patients will be randomised (1:1) into two groups: a corticosteroid injection or 12 sessions of physiotherapist-led exercise therapy. The effect of the allocated treatment will be assessed through questionnaires at 6 weeks and after 3, 6, 9, and 12 months. The primary outcome is patient's reported shoulder pain-intensity and function, measured with the Shoulder Pain and Disability Index, over 12 months follow-up. Secondary outcomes include cost effectiveness, pain-intensity, function, health-related quality of life, sleep quality, patient's global perceived effect, work absence, healthcare utilisation and adverse events. Between group differences will be evaluated using a repeated measurements analysis with linear effects models. A cost-utility analysis will be performed to assess the cost-effectiveness using quality-adjusted life years from a medical and societal perspective.

For peer review only

Ethics and dissemination

This study was approved by the Medical Ethics Committee of Erasmus MC University Medical Center Rotterdam (MEC 2020–0300). All participants will give written informed consent prior to data collection. The results from this study will be disseminated in international journals and implemented in the primary care guidelines on shoulder pain.

Article Summary

Strengths and limitations of this study

- This is a large pragmatic randomised controlled trial that aims to evaluate two treatment options recommended by the guidelines in the management of persistent shoulder pain in general practice, a corticosteroid injection compared to exercise therapy.
- In addition to the clinical effectiveness, a cost-effectiveness analysis will be performed for both treatments.
- This study has a long follow-up period of 12 months, allowing for the analysis of the long-term (cost-)effectiveness of both treatments.
- The pragmatic nature of this trial has its drawbacks, however it will provide a true reflection of both treatments applied in current practice.

Keywords: Shoulder pain; Corticosteroid injection; Exercise therapy; Primary care; General practice; Randomized clinical trial;

Trial registration

This trial is registered in the Dutch Trial Registry (number NL8854) at 2020-08-26 (https://www.trialregister.nl/trial/8854). Issue date: 30 august 2020

For peer review only

Introduction

Background and rationale

Shoulder pain is the third most common musculoskeletal complaint in primary health care [1-3]. The estimated incidence is reported at 30.3 per 1000 person-years [3]. The prognosis for shoulder pain is often unfavourable. Only 50% of people presenting with a new episode of shoulder pain in primary care show complete recovery within six months [4]. In general, apart from pain, patients with shoulder pain report having functional limitations which can reach a level of severity whereby they preclude work-related tasks [5]. Work absence and treatment of shoulder pain generate high costs to society and healthcare [6, 7]. A recent cost-estimation study for patients with shoulder pain consulting in primary care in Sweden estimated the mean annual costs at €4,139 per patient, with sick leave accounting for more than 80% of the total costs [7].

Guidelines for the management of shoulder pain provide treatment options based on the initial diagnosis of the general practitioner (GP) and the severity of the pain [8, 9]. The recommended management options in the guidelines are focused on controlling pain and restoring or maintaining shoulder function. The recently updated primary care guideline for shoulder pain, issued by the Dutch College of General Practitioners (DCGP) in 2019, recommends a stepped-care approach. In the first step, GPs are advised to start the treatment with advice and, if necessary, prescribe analgesics. If pain persists, the GP is recommended to either prolong or adjust analgesics, administer a local corticosteroid injection in case of severe pain or refer the patient to a physiotherapist for exercise therapy in case of (impending) dysfunction [8]. Although the guideline recommends exercise therapy or corticosteroid injection when shoulder pain persists, the guideline acknowledges the lack of evidence to favour one option over the other.

For peer review only

A systematic review by Steuri et al. (2017) on RCTs comparing corticosteroid injection(s) to exercise therapy showed that injections have statistically significant, but small effect on pain in the short term, generally within 6 weeks after the intervention, but not at longer follow-up time intervals (3-6 months) [10]. Given the low quality of most of the included studies and high level of heterogeneity, the authors concluded that larger, high quality trials are required. Moreover, the authors call for health economic evaluations alongside such trials to assess comparative cost-effectiveness and cost utility. A similar call came from the Cochrane review by Page et al. (2016) on manual therapy and exercise for rotator cuff disease; 'high quality RCTs are needed to establish the benefits and harms of exercise interventions that reflect actual practice, compared with placebo, no intervention or active interventions with evidence of benefit (e.g. glucocorticoid injection)' [11].

Given the high incidence and costs associated with shoulder pain and the lack of high quality evidence to underpin current clinical practice and guideline recommendations, the recently published National Research Agenda by the NHG listed research on the effectiveness of corticosteroid injections for shoulder pain in general practice as a top priority [12]. We have therefore designed a randomised controlled trial to compare the clinical and cost-effectiveness of corticosteroid injections and physiotherapist-led exercise therapy as primary care management interventions for patients with shoulder pain.

For peer review only

Objectives

The primary objective of the Shoulder Injection and eXercise (SIX) trial is to compare the clinical effectiveness of a local corticosteroid injection to physiotherapist-led exercise therapy for shoulder pain in primary care over 12 months of follow-up. The main secondary objective is to compare the cost effectiveness of both treatments over a 12 months follow-up period.

Methods and analysis

Trial design/Study setting

The study is a randomised, multicentre, open label, parallel group, pragmatic clinical trial. Patients will be recruited in Dutch general practices. GPs will select patients presented with shoulder pain who are suitable for both a local corticosteroid injection and physiotherapist-led exercise therapy. GPs will refer these patients to the SIX research team, who will further assess all potential patients for eligibility and will undertake informed consent procedures.

Eligibility criteria

Inclusion criteria:

- Patient has contacted their GP with shoulder pain due to subacromial pain syndrome or glenohumeral disorders
- Aged 18 years or older
- Qualified for both a local corticosteroid injection and physiotherapist-led exercise therapy, as indicated by the GP
- Able to understand spoken and written Dutch language

Exclusion criteria

- Shoulder pain due to recent serious trauma, malignancy, systemic rheumatologically disease, neurological or cardiac disease) [8]

For peer review only

- Shoulder pain due to instability of the glenohumeral joint, disorders of the acromio- or sternoclavicular joint, or neck pain with additional shoulder pain
- Treatment of the affected shoulder with corticosteroid injection or physiotherapy in the last 6 months
- A history of serious shoulder trauma, such as fractures, ruptures, luxation or surgery
- Contraindications for corticosteroid injection
- Current use of oral corticosteroids

For participants with bilateral shoulder pain, the most painful shoulder will be taken as the study shoulder.

Parallel cohort study

Patients with shoulder pain who are not eligible for trial participation or patients who are eligible but do not want to be randomised, e.g. due to strong treatment preferences, will be invited to participate in a parallel cohort study. With their consent, these patients will be assessed using the same outcome measures at similar time points. In addition, these patients will complete a questionnaire regarding their treatment preferences and reasons for not wanting to participate in the trial (if applicable) at baseline. This information will provide important information regarding the recruitment process by indicating if and why recruitment may be suboptimal or failing. Furthermore, the parallel cohort study will provide the unique possibility to compare baseline characteristics of randomised participants to those who were not eligible or not willing to be randomised and outcomes following their (preferred) treatment.

We anticipate recruiting around 600 patients to this parallel cohort study. All cohort participants that are not eligible for the RCT will be informed that if the initial GP treatment fails and they consider re-consultation, they are potentially eligible for the RCT. They can contact the SIX research team for receiving additional information regarding the trial and to initiate the consent procedure for the trial.

For peer review only

Interventions

Corticosteroid injection

The corticosteroid injection will be delivered by the GP. The corticosteroid injection will consists of 40 mg triamcinolone acetonide (Kenacort-A 40), possibly in combination with a local anaesthetic agent, lidocaine 10 mg, at the discretion of the GP in accordance with the NHG guideline for shoulder pain [8].

The site of the injection, subacromial or intra-articular, will depend on the initial diagnosis of the GP. Subacromial injections will be administered to participants diagnosed with subacromial pain syndrome and intra-articular injections on participants with glenohumeral joint pain. GPs are advised to follow the instructional videos on subacromial and intra-articular injection published by the NHG [13]. All participating GPs will be invited for an optional shoulder injection training by an experienced doctor of orthopaedic medicine at the Erasmus MC.

Consultations with the GP will be coordinated so that participants typically receive their injection within one week of randomisation. In line with the guideline, a maximum of 2 injections will be permitted per patient, with the second injection, when considered necessary, offered 2 to 4 weeks after the first injection. Any participant receiving a second injection will have the date of administration recorded in their case report form.

For peer review only

Physiotherapist-led exercise therapy

Participants randomized to physiotherapist-led exercise therapy will be referred to one of the local physiotherapists. Preferably the physiotherapist is affiliated with the Dutch Shoulder Network (SNN). The SNN is an umbrella organisation for regional shoulder networks of physiotherapist practices. All affiliated physiotherapists have to complete a 2-day entry course on shoulder pain, accredited by the Royal Dutch Society for Physiotherapy (KNGF).

The exercise therapy will consist of a maximum of 12 treatments of around 30 minutes under the supervision of the physiotherapist over a course of 12-14 weeks. In addition, all participants will receive home-based exercise at the discretion of the physiotherapist. The intensity of the exercise is based on tissue irritability and the capacity of the patient. Pain during or after exercise is allowed, as long as there is no night-time pain and the pain returns to pre-training levels within 24 hours. Physiotherapist will be requested not to use massage, laser therapy, ultrasound therapy, transcutaneous electrical nerve stimulation (TENS), dry needling or acupuncture, given lack of evidence for effectiveness [14]. All participating physiotherapists will receive a brief guideline developed in cooperation with the SNN describing the criteria for exercise therapy.

C*o-interventions*

This is a pragmatic clinical trial designed to evaluate the effectiveness of corticosteroid injections compared to physiotherapist-led exercise therapy for shoulder pain in real-life routine practice conditions. Therefore, participants will be instructed to continue their usual medication as discussed with their GP. Co-interventions after randomisation will be allowed and will be monitored through medical record review and questionnaires. This includes cross-over between interventions, which is estimated to occur in 20% of participants based on the number of patients receiving an injection and referral for exercise therapy in the 'usual care' treatment arm of a recent RCT [15].

For peer review only

Outcomes

Table 1 shows an overview of the time schedule of enrolment, interventions and all assessments for participants. The selection of outcome measures has been based on the core outcome set published by OMERACT [16]. The primary outcome is shoulder pain-intensity and function measured using the Shoulder Pain and Disability Index (SPADI) total score over 12 months post randomisation [17]. The SPADI is the most commonly used measure to assess pain-intensity and disability [18]. The Dutch version of the SPADI has good psychometric properties [19, 20].

Secondary outcomes include incremental costs per quality adjusted life year (QALY) gained, using both the medical as well as the societal perspective, over 12 months post randomisation. Medical costs will be measured using the Medical Cost Questionnaire (MCQ) and societal costs will be measured using the Productivity Cost Questionnaire (PCQ) [21]. QALY will be measured using the five-level version of the well-validated EuroQol Five-Dimensional Questionnaire (EQ-5D-5L) score [22].

Other secondary outcomes will be clinical- and cost effectiveness of the randomised treatments in the short term (6 weeks, 3 months) and medium term (6 months, 9 months). In addition, secondary outcomes will include subdomains (pain and function) of the SPADI, health-related quality of life (EQ-5D-5L), sleep quality measured with the Sleep Quality Scale (SQS) [23], participant's perceived recovery using the global perceived effect questionnaire [16], work absence as measured by the PCQ , healthcare utilisation as measured by the MCQ, side effects assessed at short term post randomisation and serious adverse events (SAE) occurring post randomisation.

For peer review only

Tabel 1. Time schedule for enrolment, interventions and assessments for participants.

Time-point	Pre-randomisation	Baseline (T0)	6 weeks (T1)	3 months (T2)	6-9-12 months (T3-T4-T5)[a]
**Enrolment**					
Diagnosis	X				
Eligibility screening	X				
Informed consent	X				
Randomisation[b]		X			
**Interventions**					
Corticosteroid injection[b]		X			
Physiotherapist-led exercise therapy[b]		⟵	⟶	⟶	
**Assessments**					
Socio-demographics		X			
Current shoulder episode (location, duration, cause, course, stiffness)		X			
Previous shoulder episodes (history, treatments)		X			
Other current pain locations (pain manikin)		X			
Other relevant medical issues		X			
Psychological prognostic factors (HADS, FABQ)		X			
Current medical use for the shoulder pain		X			
Treatment preferences		X			
Treatment expectations		X			
**Outcomes**					
Pain and function (SPADI)		X	X	X	X
Medical costs (MCQ)			X	X	X
Global perceived effect (GPE)			X	X	X
Productivity costs (PCQ)			X	X	X
Health-related quality of life (EQ-5D-5L)		X	X	X	X
Sleep quality (SQS)		X	X	X	X
Side effects			X	X	
Serious adverse events (SAE)			X	X	X

[a]At these time-points the indicated outcome measures will be repeated. [b]Randomisation occurs after baseline measurements are taken.

For peer review only

Sample size

The target sample size is 85 participants in each trial group. This is based on 90% power and a 0.05 two-sided statistical significance to detect a minimally clinically important difference of ten points on the SPADI total scale [24], using a conservative estimation of a baseline SD of 20 [25]. Accounting for a potential loss to follow-up at 12 months of 20%, this will require a total of 213 patients to be randomized to the intervention groups.

Recruitment

All patients (≥18 years old) consulting their GP for shoulder pain who are suitable for both a local corticosteroid injection and physiotherapist-led exercise therapy can be invited by their GP to participate in this study. These patients will be informed on the trial by the GP and are advised to contact the research team. The research team will provide further information on the trial and if the patient confirms their interest to participate in the trial, eligibility will be checked and the informed consent procedure will be completed. After the participant has completed the baseline questionnaire, the patient will be randomised by the research team. The patient and the GP will be notified on the randomisation result by the research ream.

All other patients (e.g., wait-and see policy or prescription of analgesics) will be invited to participate in the parallel cohort study. These patients will be invited through two-weekly searches of the medical records of participating GP practices. All cohort participants will be informed that if the initial GP treatment fails and they consider re-consultation, they are potentially eligible for the RCT (figure 1).

Accounting for a 25% willingness of patients to participate in the RCT and in the cohort, a 50% willingness of participants in the cohort to enter the trial if initial treatment fails, and a 25% loss due to not eligible for the trial, 2430 patients need to be invited to participate in either the trial or the cohort

For peer review only

over the 18 months period. On average a fulltime Dutch GP has around 2095 registered patients [26]. With an incidence rate of 30.3 per 1000 person-years in the Netherlands [3], it is expected that a fulltime GP sees around 6 patients with a new episode of shoulder pain per month, which results in 23 GPs needed for this study. However, taking in account Lasagna's law [27], we expect to need at least 46 GPs to ensure the total sample size.

Allocation

The Erasmus MC Clinical Trial Center, who will not meet or contact the patients will prepare a remote web-based randomization system using random blocks of 8, 6 or 4 to ensure concealment of allocation. Participants and their GP will be informed about the outcome of the randomisation by phone and participants will receive a patient card through mail detailing their treatment allocation and related procedures.

Blinding

The researcher who will carry out the primary analysis will be blinded for treatment allocation. The participant and the GP will not be blinded for treatment allocation. This is not feasible in this pragmatic trial.

Data collection methods

After obtaining informed consent, participants will complete the baseline questionnaire. Subsequently, the participants will be randomized to one of the two interventions groups. Participants will be asked to complete online questionnaires sent by e-mail, after 6 weeks, 3, 6, 9 and 12 months after randomisation. If the follow-up questionnaire is not returned within 2 weeks of initial mailing, a reminder will be sent encouraging the participant to complete the questionnaire. Non-responders or

For peer review only

responders with incomplete questionnaires will be contacted by telephone to pose them the missing questions.

Data management

Data management will be performed via a web-based medical survey tracker (Gemstracker). Each participant will be allocated a unique code, which will be used on all trial-specific documents, except for the signed informed consent and contact details. Participants' identifiable data will be stored separately and securely from study data in accordance with local procedures.

Statistical methods

Baseline characteristics will be summarized using descriptive statistics. All analyses will be performed under intention-to-treat (ITT).

Primary analyses

The primary clinical outcome is patient reported severity of pain and function over 12 months post-randomisation, measured with the SPADI total score. A linear mixed model with repeated measures will be used to generate estimates of effects. The time-points included in this model will be baseline, 6 weeks, 3, 6, 9, and 12 months. Baseline values for the primary outcome are retained as part of the outcome vector and group means on the primary outcome are assumed to be equal at baseline (i.e. an intervention-effect is restricted at baseline). Fixed effects will be time and time by treatment group. To model the covariance of repeated measures by participant, the option for data structure in the analyses will be set on 'unstructured' and the model which yields the lowest Akaike's information criterion will be chosen. The following baseline measurements will be considered as covariates: age, gender, duration of pain, concomitant neck pain and history of shoulder pain [28-30].

For peer review only

Secondary analyses

The cost effectiveness will be evaluated using the incremental cost per QALY gained of the corticosteroid injection versus physiotherapist-led exercise therapy, using both the healthcare as well as the societal perspective, using a time horizon of 12 months. Non-parametric bootstrapping will be used to depict the degree of uncertainty for costs and health effects and the cost-utility ratio in a cost-effectiveness plane. In addition, an acceptability curve will be drawn, which indicates the probability that the intervention studied has lower incremental costs per QALY gained than various thresholds for the maximum willingness to pay for an extra QALY. Similar methods will be used to estimate the cost effectiveness of both interventions in the short term (6 weeks and 3 months) and medium term (6 and 9 months).

In addition, secondary analyses include shoulder pain-intensity, shoulder function, global perceived effect, quality of life, sleep quality, work absence, healthcare utilisation and side effects and will be evaluated at all follow up time points using linear model regression methods for numerical outcomes and logistic regression methods for dichotomous outcomes. The clinical effectiveness at all other follow-up time points will be estimated using similar methods described for the primary analyses.

Subgroup analysis

Two explorative, pre-defined, subgroup analyses will be performed assessing the interaction effects between treatment and the severity of baseline pain (SPADI pain subscale) and between treatment and baseline function (SPADI function subscale) on the primary and secondary outcomes.

Sensitivity analysis

To test the robustness of the results sensitivity analysis will be performed using per-protocol principles (excluding participants with cross-over during the study period) and using complete cases only.

For peer review only

Data monitoring

This study has negligible risk according to the risk classification published in the guidelines of the Dutch Federation of University Medical Centres (NFU) [31]. Therefore, monitoring will take place once a year by independent monitors and no Data Monitoring Committee (DMC) will be assigned to this study. Trial conduct and data integrity will be audited once per year by independent auditors.

Harms

Potential adverse events will be monitored using patient self-report questionnaires, contact with the SIX research team and general practitioner reports. GPs and physiotherapists will be asked to report any serious adverse event (SAE) and suspected unexpected serious adverse reaction (SUSAR) directly to the SIX research team. The SIX research team will report the SAE or SUSAR to the the Medical Research and Ethics Committee (METC).

Patient and public involvement

Prior to the design of this trial, patients who recently consulted their GP for shoulder pain were contacted to participate in our patient panel. These patients could comment on the design and confirmed this study as relevant and feasible. The patient panel will also be used to help facilitate dissemination of the final results to trial participants and in the design of implementation strategies towards patients.

For peer review only

Ethics and Dissemination

Ethics approval and informed consent

Ethical approval on this protocol (version 3.0) was obtained on 18 September 2020 by the Medical Ethics Committee (METC) of Erasmus MC University Medical Center Rotterdam (MEC 2020–0300). Any substantial amendment made to the protocol by the coordinating investigator is sent to the METC for approval, prior to implementation. All participants will give written informed consent prior to data collection (Supplementary file).

Dissemination

Results of this trial will be published in peer-reviewed journals, as a double publication in a national general practitioners journal, to the Royal Dutch Society for Physiotherapy (KNGF), and through social media. A patient panel composed by the research team consisting of patients with shoulder pain will help facilitate the optimization of the method of dissemination of the results to participating patients. Furthermore, participating GPs and physiotherapist will be informed about trial results (expected in 2023).

Discussion

This paper presents the design of a pragmatic, randomized controlled trial that will assess the effectiveness of corticosteroid injection versus physiotherapist-led exercise therapy for shoulder pain in primary care. Furthermore, this trial will assess the cost effectiveness of both interventions from a societal and healthcare perspective. The primary outcome is shoulder pain-intensity and function measured with the SPADI over a 12 months period. Secondary outcomes are measured at 6 weeks, 3, 6, 9 and 12 months follow-up and include shoulder pain-intensity, shoulder function, global perceived

For peer review only

effect, quality of life, sleep quality, work absence, healthcare utilisation and adverse reactions. Between group differences for the primary outcome will be evaluated using a repeated measurements analysis with linear mixed models. An economic evaluation will be performed using a cost utility analysis with quality of life. The outcomes of this trial may impact the clinical guideline recommendations for the management of shoulder pain in primary care and possibly the reimbursement of physiotherapy for patients with shoulder pain. Recruitment of eligible patients is currently ongoing (November 2020). Substantial protocol amendments will be communicated to participants, cooperating GPs and physiotherapist, the Medical Research and Ethics Committee (METC), the Dutch Trial Registry, ZonMw and the journal publishing this protocol.

Acknowledgements

The authors would like to thank Gerard Koel for his contributions in the design of the exercise therapy guideline for participating physiotherapists.

Statements

Contributorship statement

JR, BK, PB, EdS, MK, RO, DS, MTdG, DvdW, JvO participated in the design of the study. EdS, PvD, JR and BK coordinate the trial and are responsible for data collection. PvD and JR prepared the article. All authors have read and approved the final manuscript.

Competing interests

The authors declare that they have no conflict of interest.

For peer review only

Funding

This trial is funded by the Netherlands Organization for Health Research and Development (ZonMw),

Grant number: 852002027

Data availability statement

Data will be available upon reasonable request. Deidentified individual participant data that will underlie the results reported in the article to be published will be shared to researcher who provide a methodologically sound proposal. Proposals should be directed to p.vandoorn@erasmusmc.nl. To gain access, data requestors will need to sign a data access agreement. Data are available beginning 3 months and ending 5 years following article publication. The data will be made available on Erasmus MC secured drivers.

For peer review only

References

1. Bot SD, van der Waal JM, Terwee CB, van der Windt DA, Schellevis FG, Bouter LM, Dekker J: **Incidence and prevalence of complaints of the neck and upper extremity in general practice**. *Ann Rheum Dis* 2005, **64**(1):118-123.
2. Greving K, Dorrestijn O, Winters JC, Groenhof F, van der Meer K, Stevens M, Diercks RL: **Incidence, prevalence, and consultation rates of shoulder complaints in general practice**. *Scand J Rheumatol* 2012, **41**(2):150-155.
3. van Doorn PF, de Schepper EIT, Rozendaal RM, Ottenheijm RPG, van der Lei J, Bindels PJ, Schiphof D: **The incidence and management of shoulder complaints in primary care: a retrospective cohort study**. *Fam Pract* 2020, **Manuscript submitted for publication**.
4. Kuijpers T, van der Windt DA, van der Heijden GJ, Bouter LM: **Systematic review of prognostic cohort studies on shoulder disorders**. *Pain* 2004, **109**(3):420-431.
5. Smith-Forbes EV, Moore-Reed SD, Westgate PM, Kibler WB, Uhl TL: **Descriptive analysis of common functional limitations identified by patients with shoulder pain**. *J Sport Rehabil* 2015, **24**(2):179-188.
6. Kuijpers T, van Tulder MW, van der Heijden GJ, Bouter LM, van der Windt DA: **Costs of shoulder pain in primary care consulters: a prospective cohort study in The Netherlands**. *BMC Musculoskelet Disord* 2006, **7**:83.
7. Virta L, Joranger P, Brox JI, Eriksson R: **Costs of shoulder pain and resource use in primary health care: a cost-of-illness study in Sweden**. *BMC Musculoskelet Disord* 2012, **13**:17.
8. **NHG Standaard Schouderklachten** [https://richtlijnen.nhg.org/standaarden/schouderklachten]
9. Codsi M, Howe CR: **Shoulder Conditions: Diagnosis and Treatment Guideline**. *Phys Med Rehabil Clin N Am* 2015, **26**(3):467-489.
10. Steuri R, Sattelmayer M, Elsig S, Kolly C, Tal A, Taeymans J, Hilfiker R: **Effectiveness of conservative interventions including exercise, manual therapy and medical management in adults with shoulder impingement: a systematic review and meta-analysis of RCTs**. *Br J Sports Med* 2017, **51**(18):1340-1347.
11. Page MJ, Green S, McBain B, Surace SJ, Deitch J, Lyttle N, Mrocki MA, Buchbinder R: **Manual therapy and exercise for rotator cuff disease**. *Cochrane Database Syst Rev* 2016(6):CD012224.
12. Nederlandse Huisartsen Genootschap: **Nationale Onderzoeksagenda Huisartsgeneeskunde**. In. www.nhg.org: NHG; 2018.
13. Nederlands Huisartsen Genootschap (NHG): **Subacromiale/Intra-articulaire injectie van de schouder**. In. portal.nhg.org; 2012.
14. Jansen MJ, Brooijmans F, Geraets JJXR, Lenssen AF, R.P.G. O, Penning LIF, de Bie RA: **KNGF evidence statement Subacromiale klachten**. In., vol. 121. www.kngfrichtlijnen.nl: Koninklijk Genootschap voor Fysiotherapie (KNGF); 2011.
15. Ottenheijm RP, Cals JW, Winkens B, Weijers RE, de Bie RA, Dinant GJ: **Ultrasound imaging to tailor the treatment of acute shoulder pain: a randomised controlled trial in general practice**. *BMJ Open* 2016, **6**(11):e011048.
16. Ramiro S, Page MJ, Whittle SL, Huang H, Verhagen AP, Beaton DE, Richards P, Voshaar M, Shea B, van der Windt DA *et al*: **The OMERACT Core Domain Set for Clinical Trials of Shoulder Disorders**. *J Rheumatol* 2019, **46**(8):969-975.
17. Roach KE, Budiman-Mak E, Songsiridej N, Lertratanakul Y: **Development of a shoulder pain and disability index**. *Arthritis Care Res* 1991, **4**(4):143-149.

For peer review only

18. Page MJ, McKenzie JE, Green SE, Beaton DE, Jain NB, Lenza M, Verhagen AP, Surace S, Deitch J, Buchbinder R: **Core domain and outcome measurement sets for shoulder pain trials are needed: systematic review of physical therapy trials**. *J Clin Epidemiol* 2015, **68**(11):1270-1281.
19. Thoomes-de Graaf M, Scholten-Peeters GG, Duijn E, Karel Y, Koes BW, Verhagen AP: **The Dutch Shoulder Pain and Disability Index (SPADI): a reliability and validation study**. *Qual Life Res* 2015, **24**(6):1515-1519.
20. Thoomes-de Graaf M, Scholten-Peeters W, Duijn E, Karel Y, de Vet HC, Koes B, Verhagen A: **The Responsiveness and Interpretability of the Shoulder Pain and Disability Index**. *J Orthop Sports Phys Ther* 2017, **47**(4):278-286.
21. Bouwmans C, Krol M, Severens H, Koopmanschap M, Brouwer W, Hakkaart-van Roijen L: **The iMTA Productivity Cost Questionnaire: A Standardized Instrument for Measuring and Valuing Health-Related Productivity Losses**. *Value Health* 2015, **18**(6):753-758.
22. M MV, K MV, S MAAE, de Wit GA, Prenger R, E AS: **Dutch Tariff for the Five-Level Version of EQ-5D**. *Value Health* 2016, **19**(4):343-352.
23. Snyder E, Cai B, DeMuro C, Morrison MF, Ball W: **A New Single-Item Sleep Quality Scale: Results of Psychometric Evaluation in Patients With Chronic Primary Insomnia and Depression**. *J Clin Sleep Med* 2018, **14**(11):1849-1857.
24. Breckenridge JD, McAuley JH: **Shoulder Pain and Disability Index (SPADI)**. *J Physiother* 2011, **57**(3):197.
25. Roddy E, Zwierska I, Hay EM, Jowett S, Lewis M, Stevenson K, van der Windt D, Foster NE, team St: **Subacromial impingement syndrome and pain: protocol for a randomised controlled trial of exercise and corticosteroid injection (the SUPPORT trial)**. *BMC Musculoskelet Disord* 2014, **15**:81.
26. Nederlandse Zorgautoriteit: **Beleidsregel huisartsenzorg en multidisciplinaire zorg**. In., 1 edn. puc.overheid.nl; 2020.
27. van der Wouden JC, Blankenstein AH, Huibers MJ, van der Windt DA, Stalman WA, Verhagen AP: **Survey among 78 studies showed that Lasagna's law holds in Dutch primary care research**. *J Clin Epidemiol* 2007, **60**(8):819-824.
28. Bot SD, van der Waal JM, Terwee CB, van der Windt DA, Scholten RJ, Bouter LM, Dekker J: **Predictors of outcome in neck and shoulder symptoms: a cohort study in general practice**. *Spine (Phila Pa 1976)* 2005, **30**(16):E459-470.
29. Engebretsen K, Grotle M, Bautz-Holter E, Ekeberg OM, Brox JI: **Predictors of shoulder pain and disability index (SPADI) and work status after 1 year in patients with subacromial shoulder pain**. *BMC Musculoskelet Disord* 2010, **11**:218.
30. Kooijman MK, Barten DJ, Swinkels IC, Kuijpers T, de Bakker D, Koes BW, Veenhof C: **Pain intensity, neck pain and longer duration of complaints predict poorer outcome in patients with shoulder pain--a systematic review**. *BMC Musculoskelet Disord* 2015, **16**:288.
31. Nederlandse Federatie van Universitair Medische Centra (NFU): **Richtlijn Kwaliteitsborging Mensgebonden Onderzoek 2019**. In.: Nederlandse Federatie van Universitair Medische Centra (NFU); March 2019.

For peer review only

Figures

Figure 1. Consort flowchart of recruitment

For peer review only

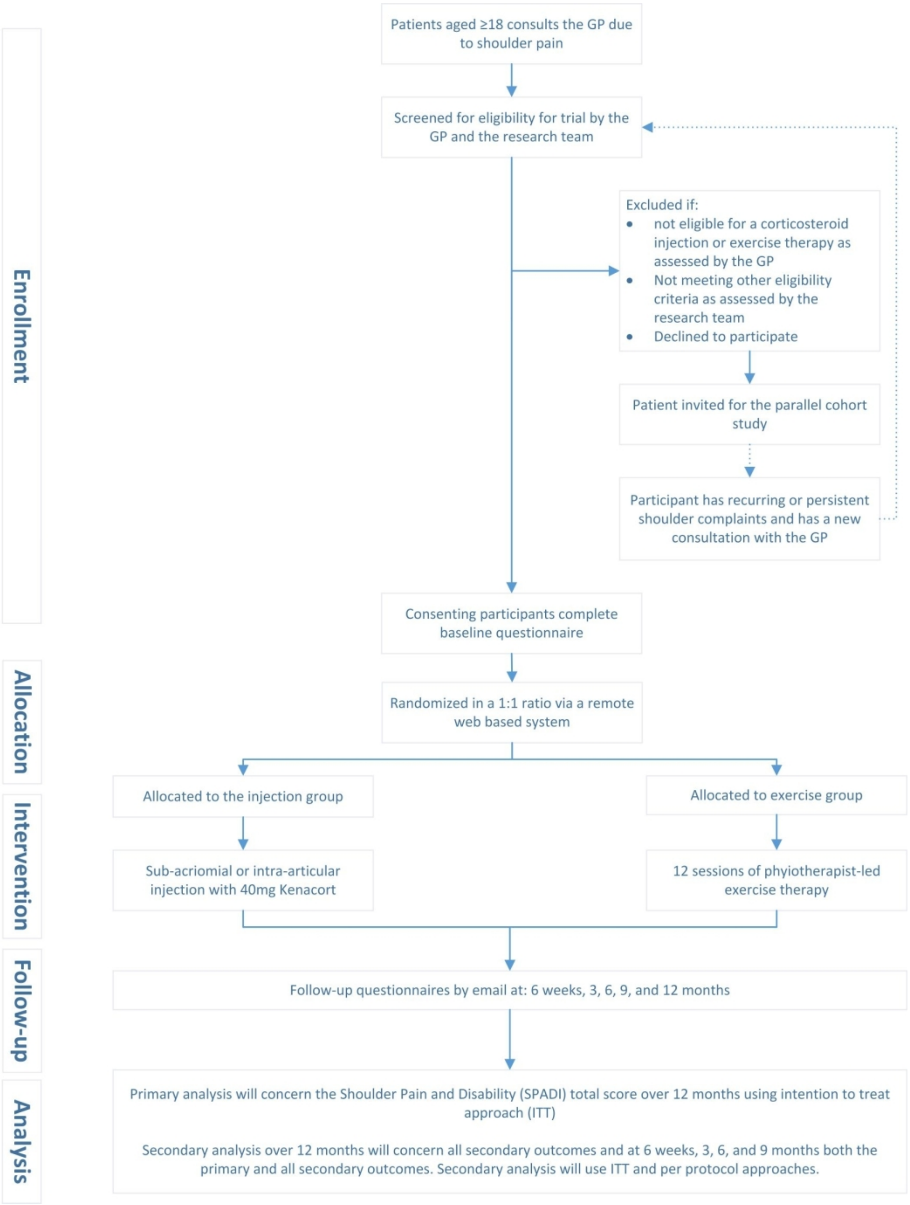

Figure 1. Consort flowchart for recruitment

36x48mm (1200 x 1200 DPI)

Bijlage C: toestemmingsformulier proefpersoon

De SIX Schouder Studie

- Ik heb de informatiebrief gelezen. Ook kon ik vragen stellen. Mijn vragen zijn voldoende beantwoord. Ik had genoeg tijd om te beslissen of ik meedoe.
- Ik weet dat meedoen vrijwillig is. Ook weet ik dat ik op ieder moment kan beslissen om toch niet mee te doen of te stoppen met het onderzoek. Daarvoor hoef ik geen reden te geven.
- Ik geef toestemming voor het informeren van mijn huisarts dat ik meedoe aan dit onderzoek.
- Ik geef toestemming voor het verzamelen en gebruiken van mijn gegevens voor de beantwoording van de onderzoeksvraag in dit onderzoek.
- Ik weet dat voor de controle van het onderzoek sommige mensen toegang tot al mijn gegevens kunnen krijgen. Die mensen staan vermeld in deze informatiebrief. Ik geef toestemming voor die inzage door deze personen.
- Ik geef □ **wel**

  □ **geen**

  toestemming om mijn persoonsgegevens langer te bewaren en te gebruiken voor toekomstig onderzoek op het gebied van schouderklachten.
- Ik geef □ **wel**

  □ **geen**

  toestemming om mij na dit onderzoek opnieuw te benaderen voor een vervolgonderzoek.
- Ik wil meedoen aan dit onderzoek.

Naam proefpersoon:
Handtekening: Datum : __ / __ / __

Ik verklaar dat ik deze proefpersoon volledig heb geïnformeerd over het genoemde onderzoek.

Als er tijdens het onderzoek informatie bekend wordt die de toestemming van de proefpersoon zou kunnen beïnvloeden, dan breng ik hem/haar daarvan tijdig op de hoogte.

Naam onderzoeker (of diens vertegenwoordiger):
Handtekening: Datum: __ / __ / __

For peer review only

For peer review only - http://bmjopen.bmj.com/site/about/guidelines.xhtml

Reporting checklist for protocol of a clinical trial.

Based on the SPIRIT guidelines.

Instructions to authors

Complete this checklist by entering the page numbers from your manuscript where readers will find each of the items listed below.

Your article may not currently address all the items on the checklist. Please modify your text to include the missing information. If you are certain that an item does not apply, please write "n/a" and provide a short explanation.

Upload your completed checklist as an extra file when you submit to a journal.

In your methods section, say that you used the SPIRITreporting guidelines, and cite them as:

Chan A-W, Tetzlaff JM, Gøtzsche PC, Altman DG, Mann H, Berlin J, Dickersin K, Hróbjartsson A, Schulz KF, Parulekar WR, Krleža-Jerić K, Laupacis A, Moher D. SPIRIT 2013 Explanation and Elaboration: Guidance for protocols of clinical trials. BMJ. 2013;346:e7586

For peer review only

		Reporting Item	Page Number
**Administrative information**			
Title	#1	Descriptive title identifying the study design, population, interventions, and, if applicable, trial acronym	1
Trial registration	#2a	Trial identifier and registry name. If not yet registered,	2

		name of intended registry	
Trial registration: data set	#2b	All items from the World Health Organization Trial Registration Data Set	4
Protocol version	#3	Date and version identifier	17
Funding	#4	Sources and types of financial, material, and other support	22
Roles and responsibilities: contributorship	#5a	Names, affiliations, and roles of protocol contributors	22
Roles and responsibilities: sponsor contact information	#5b	Name and contact information for the trial sponsor	22
Roles and responsibilities: sponsor and funder	#5c	Role of study sponsor and funders, if any, in study design; collection, management, analysis, and interpretation of data; writing of the report; and the decision to submit the report for publication, including whether they will have ultimate authority over any of these activities	22
Roles and responsibilities: committees	#5d	Composition, roles, and responsibilities of the coordinating centre, steering committee, endpoint adjudication committee, data management team, and other individuals or groups overseeing the trial, if applicable (see Item 21a for data monitoring committee)	22

Introduction

For peer review only

Background and rationale	#6a	Description of research question and justification for undertaking the trial, including summary of relevant studies (published and unpublished) examining benefits and harms for each intervention	4
Background and rationale: choice of comparators	#6b	Explanation for choice of comparators	4-5
Objectives	#7	Specific objectives or hypotheses	6
Trial design	#8	Description of trial design including type of trial (eg, parallel group, crossover, factorial, single group), allocation ratio, and framework (eg, superiority, equivalence, non-inferiority, exploratory)	5
**Methods: Participants, interventions, and outcomes**			
Study setting	#9	Description of study settings (eg, community clinic, academic hospital) and list of countries where data will be collected. Reference to where list of study sites can be obtained	6
Eligibility criteria	#10	Inclusion and exclusion criteria for participants. If applicable, eligibility criteria for study centres and individuals who will perform the interventions (eg,	6-7

For peer review only

		surgeons, psychotherapists)	
Interventions: description	#11a	Interventions for each group with sufficient detail to allow replication, including how and when they will be administered	8-9
Interventions: modifications	#11b	Criteria for discontinuing or modifying allocated interventions for a given trial participant (eg, drug dose change in response to harms, participant request, or improving / worsening disease)	9
Interventions: adherance	#11c	Strategies to improve adherence to intervention protocols, and any procedures for monitoring adherence (eg, drug tablet return; laboratory tests)	9
Interventions: concomitant care	#11d	Relevant concomitant care and interventions that are permitted or prohibited during the trial	9
Outcomes	#12	Primary, secondary, and other outcomes, including the specific measurement variable (eg, systolic blood pressure), analysis metric (eg, change from baseline, final value, time to event), method of aggregation (eg, median, proportion), and time point for each outcome. Explanation of the clinical relevance of chosen efficacy and harm outcomes is strongly recommended	10
Participant timeline	#13	Time schedule of enrolment, interventions (including any run-ins and washouts), assessments, and visits for participants. A schematic diagram is highly recommended (see Figure)	11

For peer review only

Sample size	#14	Estimated number of participants needed to achieve study objectives and how it was determined, including clinical and statistical assumptions supporting any sample size calculations	12
Recruitment	#15	Strategies for achieving adequate participant enrolment to reach target sample size	12-13
**Methods: Assignment of interventions (for controlled trials)**			
Allocation: sequence generation	#16a	Method of generating the allocation sequence (eg, computer-generated random numbers), and list of any factors for stratification. To reduce predictability of a random sequence, details of any planned restriction (eg, blocking) should be provided in a separate document that is unavailable to those who enrol participants or assign interventions	14
Allocation concealment mechanism	#16b	Mechanism of implementing the allocation sequence (eg, central telephone; sequentially numbered, opaque, sealed envelopes), describing any steps to conceal the sequence until interventions are assigned	14
Allocation: implementation	#16c	Who will generate the allocation sequence, who will enrol participants, and who will assign participants to interventions	14

For peer review only

Blinding (masking)	#17a	Who will be blinded after assignment to interventions (eg, trial participants, care providers, outcome assessors, data analysts), and how	14
Blinding (masking): emergency unblinding	#17b	If blinded, circumstances under which unblinding is permissible, and procedure for revealing a participant's allocated intervention during the trial	14
**Methods: Data collection, management, and analysis**			
Data collection plan	#18a	Plans for assessment and collection of outcome, baseline, and other trial data, including any related processes to promote data quality (eg, duplicate measurements, training of assessors) and a description of study instruments (eg, questionnaires, laboratory tests) along with their reliability and validity, if known. Reference to where data collection forms can be found, if not in the protocol	15
Data collection plan: retention	#18b	Plans to promote participant retention and complete follow-up, including list of any outcome data to be collected for participants who discontinue or deviate from intervention protocols	15
Data management	#19	Plans for data entry, coding, security, and storage, including any related processes to promote data quality	15

For peer review only

		(eg, double data entry; range checks for data values). Reference to where details of data management procedures can be found, if not in the protocol	
Statistics: outcomes	#20a	Statistical methods for analysing primary and secondary outcomes. Reference to where other details of the statistical analysis plan can be found, if not in the protocol	15-16
Statistics: additional analyses	#20b	Methods for any additional analyses (eg, subgroup and adjusted analyses)	16-17
Statistics: analysis population and missing data	#20c	Definition of analysis population relating to protocol non-adherence (eg, as randomised analysis), and any statistical methods to handle missing data (eg, multiple imputation)	17
**Methods: Monitoring**			
Data monitoring: formal committee	#21a	Composition of data monitoring committee (DMC); summary of its role and reporting structure; statement of whether it is independent from the sponsor and competing interests; and reference to where further details about its charter can be found, if not in the protocol. Alternatively, an explanation of why a DMC is not needed	17
Data monitoring: interim analysis	#21b	Description of any interim analyses and stopping guidelines, including who will have access to these interim results and make the final decision to terminate the trial	17
Harms	#22	Plans for collecting, assessing, reporting, and managing	17

For peer review only

		solicited and spontaneously reported adverse events and other unintended effects of trial interventions or trial conduct	
Auditing	#23	Frequency and procedures for auditing trial conduct, if any, and whether the process will be independent from investigators and the sponsor	17
**Ethics and dissemination**			
Research ethics approval	#24	Plans for seeking research ethics committee / institutional review board (REC / IRB) approval	18
Protocol amendments	#25	Plans for communicating important protocol modifications (eg, changes to eligibility criteria, outcomes, analyses) to relevant parties (eg, investigators, REC / IRBs, trial participants, trial registries, journals, regulators)	18
Consent or assent	#26a	Who will obtain informed consent or assent from potential trial participants or authorised surrogates, and how (see Item 32)	18
Consent or assent: ancillary studies	#26b	Additional consent provisions for collection and use of participant data and biological specimens in ancillary studies, if applicable	n/a
Confidentiality	#27	How personal information about potential and enrolled participants will be collected, shared, and maintained in order to protect confidentiality before, during, and after the	18

For peer review only

		trial	
Declaration of interests	#28	Financial and other competing interests for principal investigators for the overall trial and each study site	22
Data access	#29	Statement of who will have access to the final trial dataset, and disclosure of contractual agreements that limit such access for investigators	22
Ancillary and post trial care	#30	Provisions, if any, for ancillary and post-trial care, and for compensation to those who suffer harm from trial participation	n/a
Dissemination policy: trial results	#31a	Plans for investigators and sponsor to communicate trial results to participants, healthcare professionals, the public, and other relevant groups (eg, via publication, reporting in results databases, or other data sharing arrangements), including any publication restrictions	18
Dissemination policy: authorship	#31b	Authorship eligibility guidelines and any intended use of professional writers	n/a
Dissemination policy: reproducible research	#31c	Plans, if any, for granting public access to the full protocol, participant-level dataset, and statistical code	19
**Appendices**			
Informed consent materials	#32	Model consent form and other related documentation given to participants and authorised surrogates	See appendix
Biological specimens	#33	Plans for collection, laboratory evaluation, and storage of	n/a

For peer review only

biological specimens for genetic or molecular analysis in the current trial and for future use in ancillary studies, if applicable

The SPIRIT checklist is distributed under the terms of the Creative Commons Attribution License CC-BY-ND 3.0. This checklist was completed on 05. February 2021 using https://www.goodreports.org/, a tool made by the EQUATOR Network in collaboration with Penelope.ai

For peer review only
