# Supplementary material for: Clinical and cost effectiveness of a corticosteroid injection versus exercise therapy for shoulder pain in general practice: protocol for a randomised controlled trial (SIX Study)
Source: BMJ Open. 2021 Mar 30;11(3):e050101. doi: 10.1136/bmjopen-2021-050101 (PMC8011792; doi:10.1136/bmjopen-2021-050101)
Supplement: Reviewer comments [file bmjopen-2021-050101.reviewer_comments.pdf]

PEER REVIEW HISTORY

BMJ Open publishes all reviews undertaken for accepted manuscripts. Reviewers are asked to complete a checklist review form (**http://bmjopen.bmj.com/site/about/resources/checklist.pdf**) and are provided with free text boxes to elaborate on their assessment. These free text comments are reproduced below.

ARTICLE DETAILS

**TITLE (PROVISIONAL)**	The clinical and cost effectiveness of a corticosteroid injection versus exercise therapy for shoulder pain in general practice: Protocol for a randomized controlled trial (SIX study)
**AUTHORS**	van Doorn, Pieter; de Schepper, E; Schiphof, Dieuwke; Ottenheijm, Ramon; Thoomes-de Graaf, Marloes; Koopmanschap, M.; van Ochten, J.M.; van der Windt, Danielle; Bindels, Patrick; Koes, Bart; Runhaar, Jos

This article was not externally reviewed at BMJ Open. Protocols that have been independently assessed prior to submission to BMJ Open are usually fast-tracked to publication on the grounds that further substantial changes will not be possible. This independent assessment will usually be external, independent review for both a substantial grant award from a non-commercial or government funder and ethics approval.
